# Mathematical Modeling of COVID-19 Control and Prevention Based on Immigration Population Data in China: Model Development and Validation

**DOI:** 10.2196/18638

**Published:** 2020-05-25

**Authors:** Qiangsheng Huang, Yu Sunny Kang

**Affiliations:** 1 Ping An Technology (Shenzhen) Co, Ltd Shanghai China; 2 School of Health and Human Services University of Baltimore Baltimore, MD United States

**Keywords:** COVID-19, 2019-ncov, epidemic control and prevention, epidemic risk time series model, incoming immigration population, new diagnoses per day

## Abstract

**Background:**

At the end of February 2020, the spread of coronavirus disease (COVID-19) in China had drastically slowed and appeared to be under control compared to the peak data in early February of that year. However, the outcomes of COVID-19 control and prevention measures varied between regions (ie, provinces and municipalities) in China; moreover, COVID-19 has become a global pandemic, and the spread of the disease has accelerated in countries outside China.

**Objective:**

This study aimed to establish valid models to evaluate the effectiveness of COVID-19 control and prevention among various regions in China. These models also targeted regions with control and prevention problems by issuing immediate warnings.

**Methods:**

We built a mathematical model, the Epidemic Risk Time Series Model, and used it to analyze two sets of data, including the daily COVID-19 incidence (ie, newly diagnosed cases) as well as the daily immigration population size.

**Results:**

Based on the results of the model evaluation, some regions, such as Shanghai and Zhejiang, were successful in COVID-19 control and prevention, whereas other regions, such as Heilongjiang, yielded poor performance. The evaluation result was highly correlated with the basic reproduction number (R_0_) value, and the result was evaluated in a timely manner at the beginning of the disease outbreak.

**Conclusions:**

The Epidemic Risk Time Series Model was designed to evaluate the effectiveness of COVID-19 control and prevention in different regions in China based on analysis of immigration population data. Compared to other methods, such as R_0_, this model enabled more prompt issue of early warnings. This model can be generalized and applied to other countries to evaluate their COVID-19 control and prevention.

## Introduction

The first case of coronavirus disease (COVID-19) was diagnosed in December 2019 in Wuhan, Hubei, China. Despite the spread of COVID-19, few prevention actions were reinforced at the beginning of the disease outbreak in China. For example, a celebration banquet with tens of thousands of people was held in Wuhan on January 18, 2020; this event accelerated the spread of COVID-19 in that region [1]. Gradually, more prevention actions were taken, including investigation and control of incoming immigration populations from other regions; closing some densely populated areas; and requiring face masks to be worn in public [2,3].

In addition to the traditional methods of COVID-19 prevention and control, supplemental measures are considered to be necessary, particularly to address the issue of people who have no symptoms but may be infectious during the incubation period [4]. Specifically, the screening mechanism of taking people’s temperature before they enter public areas can only detect some COVID-19 cases [4].

Given the recent pandemic development, limited studies have utilized COVID-19-related data to investigate the effectiveness of COVID-19 control and prevention [5]. Some studies have collected media reports regarding COVID-19 to examine the role that the media has played in the current epidemic in China [6]. Similarly, researchers previously investigated norovirus epidemics via internet surveillance and built a model to predict potential disease infections in China [7].

The effectiveness of epidemic prevention and control can be estimated from statistical data, such as the daily number of newly diagnosed patients in the provinces or municipalities of China [8-10]. However, this method does not evaluate the effectiveness of prevention and control in regions (including provinces or municipalities) of China because the newly diagnosed case data are not analyzed in combination with the immigration population information during the outbreak. For example, when comparing two provinces A and B with the same numbers of newly diagnosed patients during the outbreak period, the new cases in Province A may mainly immigrate from outside the province, and most of these cases may be confirmed on the day of entrance; meanwhile, Province B may mainly consist of local residents, and most incoming cases may be confirmed one week after their entrance. All confirmed cases in both Province A and B are quarantined until being diagnosed. Therefore, the epidemic prevention and control measures in Province A should be considered to be more effective than those in Province B because the virus spread more severely in Province B despite its lower number of immigrating residents.

The Chinese government has been emphasizing the analysis of big data, especially immigration population data, in COVID-19 prevention and control since mid-February 2020 [11,12]. Immigration population data analysis is an approach to disease prevention. Particularly, the Health Code app was created [13] and applied in various regions [14-17]. The Health Code is a mobile application that detects individuals’ prior travel histories, such as in epidemic zones, before they enter a public area. Hence, to detect infected individuals prior to their entrance into public areas, it is more effective to combine this mobile application with body temperature measurements.

Several reports have analyzed the trend of population movement during the COVID-19 pandemic based on immigration population data from Baidu, Inc [18,19]. However, at present, very few COVID-19 control and prevention studies have used the dataset of the daily incoming immigration population in each region.

In this study, we analyzed immigration population data to evaluate the risk posed by the daily incoming immigration population in various regions of China. The risk output presents similar indications to the Health Code app, which evaluates the immigration risk from relevant data sources. Moreover, we built an Epidemic Risk Time Series Model to evaluate the effectiveness of COVID-19 control and prevention across different regions. Using this evaluation, regions with poor prevention performance can be detected as soon as possible.

## Methods

### Overview

**Figure 1:**
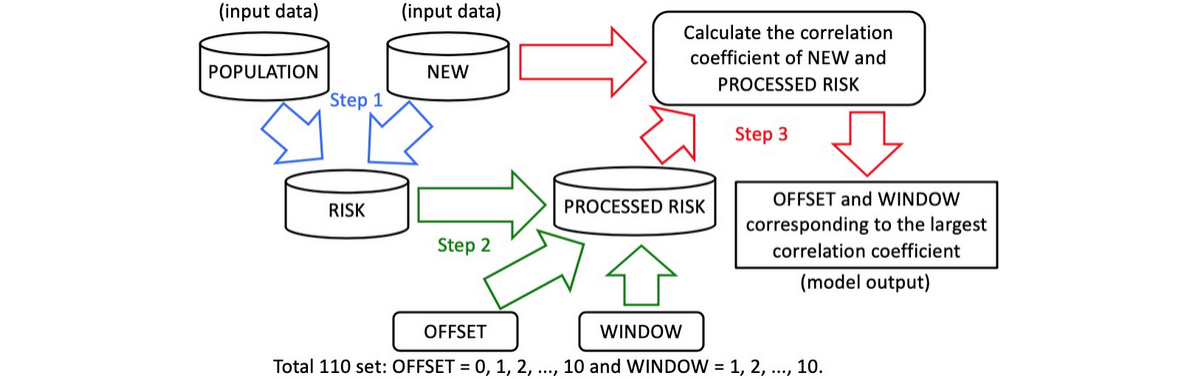
Flowchart showing the three steps of the Epidemic Risk Time Series Model.

In the Epidemic Risk Time Series Model, two decision variables, the OFFSET and WINDOW parameters, were used to reveal the delayed days of the risk (RISK) of the daily incoming immigration population (POPULATION) in each region (REGION) converting to new cases (NEW). More days indicates less effective disease control and prevention. The model workflow is shown in Figure 1. According to this model, there were three major steps to evaluate a REGION in a period of days. Specifically, first, the RISK data were constructed from POPULATION and NEW data; second, the RISK data were processed into PROCESSED RISK data using the OFFSET and WINDOW variables; last, the OFFSET and WINDOW variables that yielded the highest correlation coefficients of NEW and PROCESSED RISK data were chosen as the outputs of the model.

### Data Sources

#### Model Input Data 1: NEW

Since January 17, 2020, various REGIONs have released NEW data. The NEW data were crawled from [8].

#### Model Input Data 2: POPULATION

To detect ongoing trends of the COVID-19 epidemic, the daily incoming immigration population data, which were distinguished from different source REGIONs, were crawled from [20]. Since there were no data sources regarding immigration population data in regions such as Hong Kong, Macao, and Taiwan, and interstate traffic from Hubei has been shut down since late January, these regions were excluded from this analysis. However, the immigration populations emigrating from Hubei to other REGIONs were included in this study. All statistical analyses were conducted using Python version 3.7 (Python Software Foundation).

### Analytical Methods

#### Calculation of RISK

Incoming immigration populations of the same size exposed to different factors were at different levels of risk of contracting COVID-19. For example, individuals with prior residence in Hubei during the spread of COVID-19 experienced higher risks of being infected than individuals in other immigration populations with the same size. Hence, POPULATION was processed using Equation 1, and the RISK data were constructed.


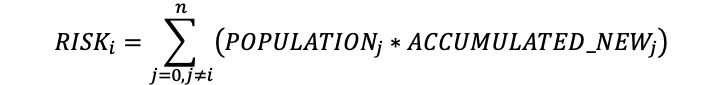


In Equation 1, all the values of RISK, POPULATION, and ACCUMULATED NEW are for a single day. The RISK_i_ value is the daily immigration risk of REGION i in one day. i can be 1, 2, 3, …, n, where n is a fixed number. In this study, n was 31 because we analyzed 31 REGIONs, including Hubei. The i value in this study cannot be the number of Hubei for the reason mentioned. The POPULATION_j_ was the POPULATION of source REGION j, where j can be 1, 2, 3, …, n, and j cannot be the same as i. ACCUMULATED_NEW_j_ was the sum of NEW in immigration source REGION j in the last 3 days (ACCUMULATED NEW), and it was calculated with Equation 2. ACCUMULATED_NEW_d_ is the ACCUMULATED NEW value on date d.

*ACCUMULATED_NEW_d_* = *NEW_d_* + *NEW_d-1_* + *NEW_d-2_*

#### OFFSET

The OFFSET variable was used to evaluate the control of the incoming immigration population. Among the incoming immigration populations, disease control and prevention were varied at different times or in different regions. Specifically, some regions implemented strict screening mechanisms, such as measuring temperature and examining cough symptoms, to detect infected immigrants and to reinforce quarantine immediately. Therefore, NEW increased simultaneously with the sudden increase of RISK on the same day, whereas infected individuals were diagnosed and confirmed relatively late if they had been infected before entering the REGION. The OFFSET was the number of days that the RISK was shifted. For example, if OFFSET was 3, the RISK of each day was processed as the RISK of 3 days ago.

#### WINDOW

The WINDOW variable was used to evaluate the control for domestic/local residents. The control and spread among the local people as well as their awareness of prevention would affect the spread of the epidemic. In some regions, immigrants were strictly home-quarantined for 14 days [21]. These rigorous measures prevented potentially infected people from spreading the virus when entering that region.

According to this model, hypothetically, when only deals with externally infected individuals, there will only include the OFFSET. On the other hand, other conditions may contribute to the spread of COVID-19 and have prolonged impact on the RISK. For instance, an infected individual who travels to the REGION, whether sick or incubating the virus, may not seek immediate medical treatment; also, local residents may have poor disease awareness and may not wear a face mask in public areas. Therefore, the WINDOW concept was introduced to the model. For example, when the WINDOW is 10, the total RISK of 10 consecutive days will affect the NEW value on the 10th day. Moreover, the incubation period with a 95% confidence interval was between 4.1 and 7.0 days. Hence, the infected person who entered the REGION 10 days ago could still affect the REGION by spreading the disease from person to person [22].

#### Processing RISK by OFFSET and WINDOW

RISK can be processed by OFFSET and WINDOW, as in Equation 3.


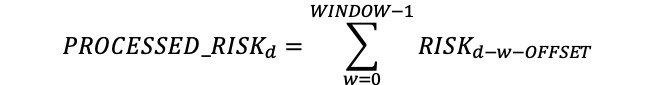


In Equation 3, all the PROCESSED RISK and RISK values are for the same REGION. PROCESSED_RISK_d_ is the value of PROCESSED RISK by OFFSET and WINDOW on date d. RISK_d-w-OFFSET_ is the value of RISK on the date d-w-OFFSET. Specifically, if it is necessary to calculate the value of PROCESSED RISK on February 11, 2020, when OFFSET is 3, WINDOW is 2. The equation is as follows:

*PROCESSED_RISK_02/11/2020_* = *RISK_02/08/2020_* + *RISK_02/07/2020_* (if OFFSET = 3, WINDOW = 2)

When OFFSET equals 0, WINDOW is 1. PROCESSED_RISK_d_ is simply RISK_d_ without any process:

*PROCESSED_RISK_d0_* = *RISK_d_* (if OFFSET = 0, WINDOW = 1)

#### Correlation Coefficients Between NEW and PROCESSED RISK and Model Outputs

The final step of this model was to find a set of OFFSET and WINDOW that was the best fit for the NEW and PROCESSED RISK values of each REGION on a daily basis.

For each REGION on a daily basis, starting from January 17, 2020, which was the first day of NEW data collection, the OFFSET was calculated from 0 to 10 and the WINDOW was calculated from 1 to 10. There were 110 different OFFSET and WINDOW sets, and the 110 sets were used to process RISK accordingly to calculate the 110 correlation coefficients with NEW and PROCESSED RISK. Finally, the set of OFFSET and WINDOW data corresponding to the maximum correlation coefficient (CORR) was the model output for the REGION on that day.

## Results

### Processing POPULATION and NEW Into RISK

Based on Equation 2, ACCUMULATED NEW was processed from NEW. As an example, the process for Hubei in the first 6 days is shown in Table 1. Accurate data were released starting on January 17; the values before that day were set to 0. Similar calculations were performed in the other 30 REGIONs on a daily basis.

**Table 1:** The NEW and ACCUMULATED NEW data in Hubei Province from January 17-22, 2020.

Date	NEW in Hubei	ACCUMULATED NEW in Hubei
01/17/2020	17	17
01/18/2020	59	76
01/19/2020	77	153
01/20/2020	72	208
01/21/2020	105	254
01/22/2020	69	246

**Figure 2:**
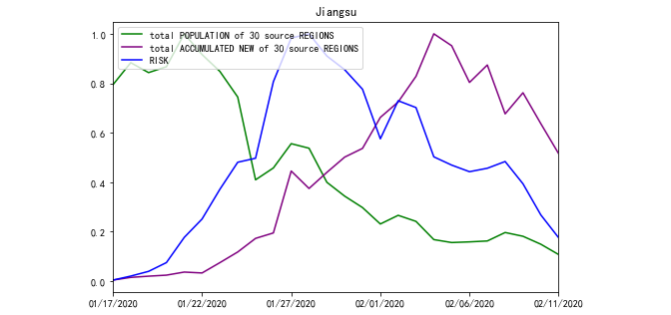
RISK of Jiangsu Province from January 17, 2020 to February 11, 2020.

**Figure 3:**
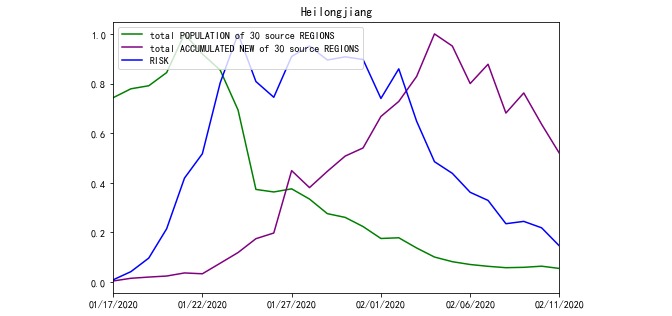
RISK of Heilongjiang Province from January 17, 2020 to February 11, 2020.

Based on Equation 1, RISK was processed from POPULATION and ACCUMULATED NEW. For example, the total POPULATION travelling to Jiangsu and Heilongjiang Province and the total ACCUMULATED NEW of source REGIONS on a daily basis are compared with their RISK in Figure 2 and Figure 3. Meanwhile, according to Equation 1, there were 30 incoming POPULATION and ACCUMULATED NEW values for every targeted REGION. To avoid plotting too many polylines in the chart, the total POPULATION and ACCUMULATED NEW polylines were plotted.

Moreover, we only analyzed the correlation among the 3 variables POPULATION, ACCUMULATED NEW, and RISK within the same region; therefore, we merged the effects and set the range of the 3 lines to zero and one.

### PROCESSED RISK and the Correlation Coefficient

**Figure 4:**
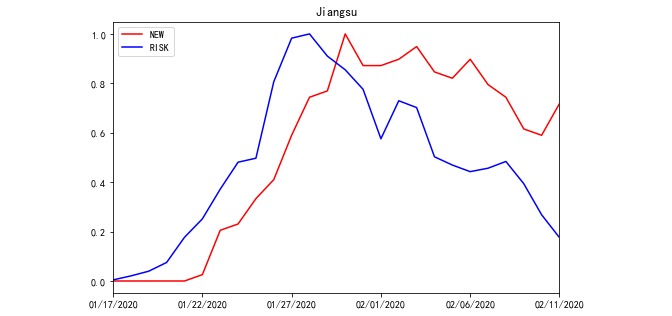
The polyline chart of NEW and RISK for Jiangsu Province from January 17, 2020 to February 11, 2020.

**Figure 5:**
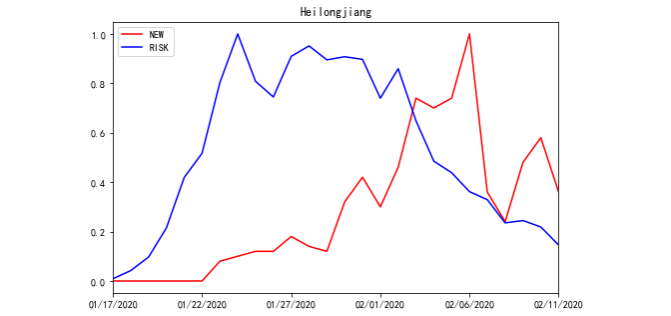
The polyline chart of NEW and RISK for Heilongjiang Province from January 17, 2020 to February 11, 2020.

**Figure 6:**
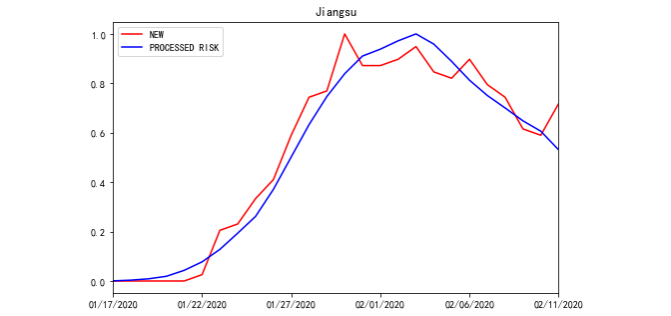
The polyline chart of NEW and PROCESSED RISK for Jiangsu Province from January 17, 2020 to February 11, 2020 when OFFSET=0 and WINDOW=9.

**Figure 7:**
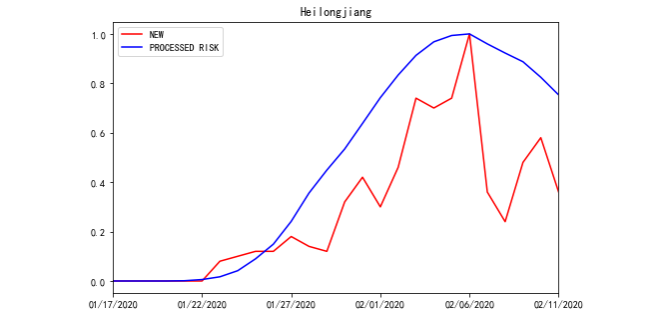
The polyline chart of NEW and PROCESSED RISK for Heilongjiang Province from January 17, 2020 to February 11, 2020 when OFFSET=4 and WINDOW=10.

In each REGION, 110 sets of OFFSET and WINDOW data were used to generate RISK on a daily basis. Due to the large amounts of data, line charts of the NEW, RISK, and PROCESSED RISK processed by the model outputs in Jiangsu and Heilongjiang from January 17 to February 11 are used here to illustrate the roles of the OFFSET and WINDOW parameters. As illustrated in Figures 4 and 5, only the absolute values of NEW and RISK were collected from the same REGION to calculate the relative indices. Hence, we defined the range of variable values to be between 0 and 1. The correlation coefficients between NEW and RISK of Jiangsu and Heilongjiang were 0.684 and –0.014, respectively. The value of Jiangsu was not high, and that of Heilongjiang was nearly uncorrelated (Figures 4 and 5). When we used PROCESSED RISK instead of RISK to draw the polyline chart, the polylines were more fitted, as illustrated in Figures 6 and 7. The correlation coefficient values increased to 0.979 and 0.874, respectively (Figures 6 and 7).

As illustrated in Figures 4-7, the OFFSET and WINDOW variables revealed the delayed days before RISK converted to NEW. In theory, if all infected individuals entering the REGION could be immediately detected and quarantined, the polylines of NEW and RISK would be fully fitted. Moreover, under this condition, the value of OFFSET would be 0, that of WINDOW would be 1, and that of CORR would be 1. On the other hand, if the infected people entering the REGION were not detected promptly and spread the virus after entering, RISK would affect NEW in the next few days. The delayed days were evaluated by the values of OFFSET and WINDOW.

### Model Output

The original size of the dataset was large; therefore, we only included the sample results from every three days between January 21, 2020 and February 11, 2020 from 11 REGIONs (Table 2), which were compared to actual data released from news reports. The study period was chosen based on the severity of the COVID-19 spread in China: the early spread of COVID-19 from Hubei Province to other REGIONs in the country to NEW was gradually decreasing in most of the REGIONs. The complete outputs are included as an appendix to this paper (Multimedia Appendix 1).

Table 2 can be used to evaluate the effectiveness of the COVID-19 control and prevention efforts in each REGION on a daily basis. The value NN indicates no confirmed cases in the REGION during the study period. The REGIONs were sorted by the values of OFFSET+WINDOW on February 11, 2020 in ascending order, which also indicated the sorting order of control and prevention effectiveness. Based on the evaluation results, Shanghai presented the lowest OFFSET and WINDOW values among the 11 REGIONs, which indicated the highest effectiveness in COVID-19 control and prevention. In contrast, Heilongjiang was the least effective REGION in COVID-19 control and prevention.

### Confirmation of the Model Outputs With Related News Reports

Limited data has been released that can be used to compare the effectiveness of disease control and prevention in the different REGIONs. However, we were able to collect data and news reports from 11 REGIONs to compare and confirm the model outputs.

First, according to the data released by the DXY Doctor Network up to February 11, 2020, the cumulative confirmed cases were grouped by incoming immigrants and local residents from three REGIONs: Shanghai, Beijing, and Tianjin (Table 3).

We then compared the cumulative confirmed cases with the OFFSET and WINDOW values in Table 2. Shanghai generated the lowest OFFSET+WINDOW value, and it performed best in COVID-19 control and prevention; also, the local residents’ infection rate in Shanghai was the lowest among the REGIONs. Beijing ranked second in performance evaluation. Tianjin demonstrated the highest OFFSET+WINDOW value; therefore, it ranked the lowest in performance (Table 2).

Second, basic reproduction number (R_0_) data for Shanghai, Zhejiang, Sichuan, Jiangsu, Henan, and Anhui were collected [23]. The R_0_ values on February 10, 2020, are shown in Table 4. Compared with Table 2, the relative values and rankings of R_0_ and OFFSET+WINDOW during the time around February 10, 2020 are nearly identical.

**Table 2:** OFFSET (O) and WINDOW (W) values from 11 REGIONs between January 21, 2020 and February 11, 2020.

	01/21		01/24			01/27			01/30			02/02			02/05			02/08			02/11	
REGION	O	W	O	W	O		W	O		W	O		W	O		W	O		W	O		W
Shanghai	3	1	3	1	6		1	6		1	1		1	0		3	1		1	0		1
Liaoning	NN^a^	NN	7	3	1		1	1		1	1		1	1		1	1		1	1		1
Zhejiang	4	1	2	1	2		1	2		1	1		2	1		2	1		3	1		3
Beijing	1	2	2	1	4		2	0		1	0		8	1		4	1		3	0		5
Jilin	NN	NN	2	1	0		1	2		1	8		9	6		6	6		3	6		1
Tianjin	4	1	1	1	1		1	0		1	6		1	6		1	6		1	6		2
Sichuan	NN	NN	1	4	2		1	0		10	1		6	1		6	1		6	1		7
Jiangsu	NN	NN	2	1	1		4	1		4	0		6	0		7	0		8	0		9
Anhui	NN	NN	3	2	0		10	4		2	2		10	3		10	3		10	3		10
Henan	4	1	6	7	4		1	2		10	2		10	4		10	3		10	3		10
Heilongjiang	NN	NN	2	5	0		10	0		10	3		10	6		10	4		10	4		10

^a^NN: values indicate no confirmed diagnosis until that day.

**Table 3:** Confirmed cases and infection rates in incoming immigrants and local residents in three of the studied REGIONs.

REGION	Incoming immigrants (n)	Local residents (n)	Local infection rate (%)
Shanghai	99	207	67.6
Beijing	25	352	93.4
Tianjin	6	106	94.6

**Table 4:** Basic reproduction numbers for 6 REGIONs on February 10, 2020.

REGION	R_0_^a^ value
Shanghai	0.46
Zhejiang	0.52
Sichuan	0.81
Jiangsu	0.82
Henan	0.75
Anhui	0.98

^a^R_0_: basic reproduction number.

The model output was confirmed by related news outlets as follows: in late January, a large group of infected businessmen returned to Wenzhou, Zhejiang from Wuhan, Hubei [24]. On February 1, 2020, the municipal government of Wenzhou, Zhejiang issued 25 control and prevention measures in a timely manner [25,26]. On February 22, 2020, after the Wenzhou epidemic was completely under control, the Chinese government newspaper published an article strongly affirming Wenzhou’s achievements in epidemic control and prevention [27]. With the outbreak of COVID-19 in Wenzhou City, its province, Zhejiang, performed well in COVID-19 control and prevention. Our model confirmed this evaluation result by presenting relatively low values of OFFSET and WINDOW in Zhejiang (Table 2).

In addition, according to survey data, Heilongjiang did not pay sufficient attention to the epidemic and showed poor prevention awareness [28]. This was also confirmed by our study results, with high OFFSET and WINDOW values (Table 2). Particularly, an online survey was conducted on January 31, 2020 that targeted 10,304 residents of three provinces in Northeastern China, namely Heilongjiang, Jilin, and Liaoning. This survey examined people’s feelings of being “confident,” “alert,” and “scared” during the COVID-19 outbreak. The level of feeling was ranked between 0 and 5, with 5 being the strongest feeling. Based on this survey, Heilongjiang demonstrated the lowest level of awareness of disease control and prevention; meanwhile, Liaoning demonstrated the highest level, and Jilin ranked second in awareness (Table 5). The survey results were also confirmed by our model (Table 2).

**Table 5:** Online survey results regarding awareness of COVID-19 control and prevention from three provinces in northeastern China. Participants ranked their feelings from 0-5, where 5 was the strongest feeling.

	Feelings toward the COVID-19^a^ outbreak		
REGION	Confident	Alert	Scared
Heilongjiang	4.1	3.8	2.1
Jilin	3.9	3.9	2.2
Liaoning	3.7	3.9	2.3

^a^COVID-19: coronavirus disease.

Using the hypothesis-testing approach described above [29], the data in Table 2, Table 3, Table 4, and Table 5 were tested based on our hypotheses. First, the correlation coefficient between the internal infection rates of the three REGIONs in Table 3 and the corresponding OFFSET+WINDOW values of these three REGIONs in Table 2 on February 11 was 0.9216, and the original hypothesis H_0_, the correlation between the local infection rate and the OFFSET+WINDOW value, was not statistically significant. For the alternative hypothesis H_a_, the correlation coefficient between the local infection rate and the OFFSET+WINDOW value was correlated; we obtained a *t* value of 2.374, and the two-tailed *P* value was .254.

The correlation coefficient between the R_0_ values of the six REGIONs in Table 4 and the corresponding OFFSET+WINDOW values of these 6 REGIONs in Table 2 on February 11, 2020 was 0.8787. The original hypothesis H_0_ assumed that the correlation between the R_0_ value and OFFSET+WINDOW value was not statistically significant; meanwhile, the alternative assumption was that the H_a_:R_0_ value and OFFSET+WINDOW value were correlated. The *t* value was calculated to be 3.682, aand the two-tailed *P* value was .021.

Finally, for the three REGIONs in Table 5, the correlation coefficient between the “alert+scared–confident” values and the corresponding “OFFSET+WINDOW” values for the three REGIONs in Table 2 on February 11 was –.9999. Specifically, the sizes of the alert and scared values were correlated to the “alert,” so the correlation coefficient was positive; meanwhile, the “confidence” and “alert” values were inversely correlated, so the correlation coefficient was negative. In addition, we proposed the hypothesis H_0_ that the correlation coefficient between the “alert+scared–confident” value and the OFFSET+WINDOW value was not significant. The alternative hypothesis H_a_ was that the “alert+scared–confident” value and the OFFSET+WINDOW value were correlated; the *t* value was –73.32 and the two-tailed *P* value was .009.

In summary, based on the 3 *P* values, the model results were highly correlated with the three datasets; this confirmed the validity of the model.

## Discussion

### Principal Findings

In this paper, the effectiveness of COVID-19 outbreak control and prevention across China was evaluated using population movement data between regions and daily new confirmed cases. Moreover, the comparison of the model output (Table 2) through the infection rate among local residents (Table 3), R_0_ value (Table 4), and vigilance survey (Table 5) confirmed the correctness of the Epidemic Risk Time Series Model; that is, when a region was evaluated by the model to perform better in control and prevention, the R_0_ value was smaller, the infection rate of local residents was lower, and residents’ vigilance regarding the COVID-19 outbreak was stronger.

### Early Warning by the Epidemic Risk Time Series Model in Epidemic Control and Prevention

According to Figure 5, the peak day of new cases (NEW) in Heilongjiang was February 6, 2020. The peak day of RISK in Heilongjiang was January 24, 2020, which was 13 days prior to the peak day of NEW. Based on Table 3, the values of OFFSET and WINDOW in Heilongjiang rose gradually from the first day. Therefore, the current daily incidence (newly diagnosed cases) could have been lower in Heilongjiang if the control and prevention measures had been stricter in Heilongjiang from the end of January 2020.

Based on our model, the warning threshold should be triggered as “problematic” when the value of OFFSET+WINDOW is ≥ 5 (Table 3); when the combined value of OFFSET+WINDOW is ≥ 10, the situation should be considered “serious.” The warning level may be affected by factors such as the incubation period. Hence, when this model is used to evaluate the effectiveness of control and prevention for other epidemics, the warning values should be modified accordingly.

### The Epidemic Risk Time Series Model vs the R_0_ Method

Compared to the R_0_ evaluation method [23], the Epidemic Risk Time Series Model was able to detect the “warning threshold” more promptly. For example, the first confirmed case in Heilongjiang was diagnosed on January 23, 2020. According to our model, the OFFSET+WINDOW values of Heilongjiang on that day were 6 and 7; the value of OFFSET+WINDOW continued to increase gradually since then (Table 3). On the other hand, the R_0_ method can only be used at least 5 days after the first confirmed cases in that REGION, which is the average incubation period [22].

### The Formula for Calculating RISK

In Equation 2, the “recent 3 days” in ACCUMULATED NEW_j_ is derived from the following considerations. Based on this study, the lower the number of days used in the calculation, the greater the CORR value generated in the later step of the model. The number of diagnoses after a long-term incubation period did not readily reflect the current RISK from its original REGION. The NEW value may vary greatly on a daily basis. Moreover, the days of suspected cases converting into confirmed cases may vary by day. Therefore, “recent 3 days” was used to calculate RISK in this model.

In Equation 1, we categorized the total population of the source REGION before calculating the cumulative cases. The values of ACCUMULATED NEW_j_ grouped by the two source REGIONs were equal. Particularly, the people in a REGION with a smaller population size presented greater probability than the infected patients traveling to the destination REGION. The CORR values remained constant, whereas the values of OFFSET+WINDOW increased to fit similar CORR values. Compared to local residents, immigrating individuals were more likely to be infected with the virus. Hence, Equation 1 was used when calculating RISK.

### Conclusion

In this study, a mathematical model was built using the number of daily confirmed cases and the daily immigration population size; the effectiveness of epidemic control and prevention, evaluated by OFFSET+WINDOW, were the outputs of the model. The results indicated that the OFFSET+WINDOW values may change daily with effective control and prevention. For REGIONs with poor performance, warning systems were triggered by the OFFSET+WINDOW values 2 weeks prior to their peak days of cases. Compared to the R_0_ method, the Epidemic Risk Time Series Model is more prompt in aiding disease control and prevention.

Although the POPULATION data may have different statistical units in other countries, we utilized the relative values of the POPULATION to calculate the correlation coefficient. Therefore, the model does not only apply to Chinese data. Theoretically, the method in this study can be generalized to other countries to evaluate the effectiveness of their COVID-19 control and prevention measures.

## Appendix

Multimedia Appendix 1 Additional input and output data and figures for the Epidemic Risk Time Series Model.

## Abbreviations

CORR: correlation coefficient
COVID-19: coronavirus disease
H_0_: original hypothesis
H_a_: alternative hypothesis
R_0_: basic reproduction number
