# Supplementary material for: Mathematical Modeling of COVID-19 Control and Prevention Based on Immigration Population Data in China: Model Development and Validation
Source: JMIR Public Health Surveill. 2020 May 25;6(2):e18638. doi: 10.2196/18638 (PMC7250064; doi:10.2196/18638)
Supplement: Multimedia Appendix 1 [file publichealth_v6i2e18638_app1.zip › more data and figures.html]

analysis


In [1]:

```
from coronavirus_analyzer import CoronavirusAnalyzer
from sklearn import preprocessing
import pandas as pd
import numpy as np
import datetime
pd.set_option('display.max_columns', None)
pd.set_option('display.max_rows', None)
```

In [2]:

```
first_date = '2020-01-17'
last_date = '2020-02-11'
in_english = True
mentioned_regions = ['Shanghai', 'Liaoning', 'Zhejiang', 'Beijing', 'Sichuan', 'Tianjin', 
                     'Jilin', 'Jiangsu', 'Anhui', 'Henan', 'Heilongjiang']
analyzer = CoronavirusAnalyzer(last_date=last_date, first_date=first_date, in_english=in_english)
```

```
2020-05-10 11:14:48,818 - numexpr.utils - INFO - NumExpr defaulting to 4 threads.
```

In [3]:

```
region = 'Hubei'
s_daily = analyzer.df_virus_daily_inc_injured[region]
s_cum = analyzer.get_df_virus_daily_inc_injured_cum_n(3, False)[region]
df = pd.DataFrame([s_daily, s_cum]).T.loc[:'2020-01-22']
df.columns = ['NEW in Hubei', 'ACCUMULATED NEW in Hubei']
df.index = ['{}/{}/{}'.format(date[5:7], date[-2:], date[:4]) for date in df.index]
df.to_excel('table1.xlsx')
df
```

Out[3]:

|  | NEW in Hubei | ACCUMULATED NEW in Hubei |
| --- | --- | --- |
| 01/17/2020 | 17 | 17 |
| 01/18/2020 | 59 | 76 |
| 01/19/2020 | 77 | 153 |
| 01/20/2020 | 72 | 208 |
| 01/21/2020 | 105 | 254 |
| 01/22/2020 | 69 | 246 |

In [4]:

```
# figure 2
def plot_chart(region):
    s_population = analyzer.df_curve_in.loc['2020-01-17': '2020-02-11', region]
    s_risk = analyzer.df_move_in_risk.loc['2020-01-17': '2020-02-11', region]
    df_new = analyzer.df_virus_daily_inc_injured.loc['2020-01-17': '2020-02-11']
    del df_new[region]
    s_new = df_new.sum(axis=1)

    s_population *= s_risk.max() / s_population.max()
    s_new *= s_risk.max() / s_new.max()
    max_val = s_new.max()
    s_population /= max_val
    s_new /= max_val
    s_risk /= max_val

    for s in [s_population, s_new, s_risk]:
        s.index = ['{}/{}/{}'.format(date[5:7], date[-2:], date[:4]) for date in s.index]
    
    s_population.plot(color='g', label='total POPULATION of 30 source REGIONS', figsize=(8, 5))
    s_new.plot(color='purple', label='total ACCUMULATED NEW of 30 source REGIONS', figsize=(8, 5))
    s_risk.plot(color='b', label='RISK', figsize=(8, 5))
    analyzer.plt.title(region)
    analyzer.plt.legend(loc='upper left')
    analyzer.plt.show()
plot_chart('Jiangsu')
```

In [5]:

```
# figure 3
plot_chart('Heilongjiang')
```

In [ ]:

```

```

## Data 1: daily new diagnosed in each region¶

regions sorted by the mean value of "new"

(including Hubei)

In [6]:

```
df_virus_daily_inc_injured = analyzer.df_virus_daily_inc_injured
sorted_regions = df_virus_daily_inc_injured.mean().sort_values(ascending=False).index
df_virus_daily_inc_injured[sorted_regions]
```

Out[6]:

|  | Hubei | Guangdong | Henan | Zhejiang | Hunan | Anhui | Jiangxi | Jiangsu | Chongqing | Shandong | Sichuan | Heilongjiang | Beijing | Shanghai | Fujian | Hebei | Shaanxi | Guangxi | Yunnan | Hainan | Guizhou | Shanxi | Liaoning | Tianjin | Gansu | Jilin | Neimenggu | Xinjiang | Ningxia | Qinghai | Xizang |
| --- | --- | --- | --- | --- | --- | --- | --- | --- | --- | --- | --- | --- | --- | --- | --- | --- | --- | --- | --- | --- | --- | --- | --- | --- | --- | --- | --- | --- | --- | --- | --- |
| date |  |  |  |  |  |  |  |  |  |  |  |  |  |  |  |  |  |  |  |  |  |  |  |  |  |  |  |  |  |  |  |
| 2020-01-17 | 17 | 0 | 0 | 0 | 0 | 0 | 0 | 0 | 0 | 0 | 0 | 0 | 0 | 0 | 0 | 0 | 0 | 0 | 0 | 0 | 0 | 0 | 0 | 0 | 0 | 0 | 0 | 0 | 0 | 0 | 0 |
| 2020-01-18 | 59 | 0 | 0 | 0 | 0 | 0 | 0 | 0 | 0 | 0 | 0 | 0 | 0 | 0 | 0 | 0 | 0 | 0 | 0 | 0 | 0 | 0 | 0 | 0 | 0 | 0 | 0 | 0 | 0 | 0 | 0 |
| 2020-01-19 | 77 | 1 | 0 | 0 | 0 | 0 | 0 | 0 | 0 | 0 | 0 | 0 | 0 | 0 | 0 | 0 | 0 | 0 | 0 | 0 | 0 | 0 | 0 | 0 | 0 | 0 | 0 | 0 | 0 | 0 | 0 |
| 2020-01-20 | 72 | 16 | 0 | 0 | 0 | 0 | 0 | 0 | 0 | 0 | 0 | 0 | 5 | 1 | 0 | 0 | 0 | 0 | 0 | 0 | 0 | 0 | 0 | 0 | 0 | 0 | 0 | 0 | 0 | 0 | 0 |
| 2020-01-21 | 105 | 9 | 1 | 5 | 0 | 0 | 2 | 0 | 5 | 0 | 0 | 0 | 5 | 8 | 0 | 0 | 0 | 0 | 0 | 0 | 0 | 0 | 0 | 2 | 0 | 0 | 0 | 0 | 0 | 0 | 0 |
| 2020-01-22 | 69 | 6 | 3 | 5 | 0 | 9 | 1 | 1 | 4 | 0 | 7 | 0 | 4 | 7 | 1 | 1 | 0 | 2 | 0 | 4 | 0 | 1 | 2 | 2 | 0 | 0 | 0 | 0 | 0 | 0 | 0 |
| 2020-01-23 | 105 | 21 | 5 | 33 | 24 | 6 | 4 | 8 | 18 | 9 | 8 | 4 | 12 | 4 | 2 | 1 | 3 | 11 | 0 | 4 | 3 | 0 | 1 | 1 | 2 | 3 | 1 | 2 | 2 | 0 | 0 |
| 2020-01-24 | 180 | 25 | 23 | 19 | 19 | 24 | 11 | 9 | 30 | 12 | 13 | 5 | 10 | 13 | 15 | 6 | 2 | 10 | 5 | 9 | 1 | 5 | 9 | 3 | 2 | 1 | 1 | 1 | 1 | 0 | 0 |
| 2020-01-25 | 323 | 33 | 51 | 42 | 26 | 21 | 18 | 13 | 18 | 18 | 16 | 6 | 15 | 7 | 11 | 5 | 17 | 10 | 6 | 2 | 1 | 3 | 7 | 2 | 3 | 0 | 3 | 1 | 1 | 1 | 0 |
| 2020-01-26 | 371 | 35 | 45 | 24 | 31 | 10 | 12 | 16 | 35 | 24 | 25 | 6 | 17 | 13 | 27 | 5 | 13 | 13 | 8 | 3 | 2 | 4 | 3 | 4 | 7 | 2 | 6 | 1 | 3 | 3 | 0 |
| 2020-01-27 | 1291 | 42 | 40 | 45 | 43 | 36 | 24 | 23 | 22 | 24 | 21 | 9 | 12 | 13 | 3 | 15 | 11 | 5 | 7 | 11 | 2 | 7 | 5 | 9 | 5 | 2 | 2 | 5 | 4 | 2 | 0 |
| 2020-01-28 | 840 | 53 | 38 | 123 | 78 | 46 | 37 | 29 | 15 | 34 | 18 | 7 | 22 | 14 | 23 | 15 | 10 | 7 | 25 | 10 | 0 | 7 | 9 | 1 | 5 | 1 | 3 | 3 | 1 | 0 | 0 |
| 2020-01-29 | 1032 | 70 | 72 | 132 | 56 | 48 | 53 | 30 | 18 | 24 | 34 | 6 | 12 | 21 | 19 | 17 | 7 | 20 | 19 | 3 | 3 | 8 | 3 | 3 | 2 | 5 | 2 | 1 | 5 | 0 | 1 |
| 2020-01-30 | 1220 | 82 | 74 | 109 | 55 | 37 | 78 | 39 | 41 | 33 | 35 | 16 | 18 | 27 | 19 | 17 | 24 | 9 | 6 | 3 | 3 | 4 | 6 | 4 | 3 | 0 | 2 | 3 | 4 | 2 | 0 |
| 2020-01-31 | 1347 | 127 | 70 | 62 | 57 | 60 | 46 | 34 | 32 | 24 | 30 | 21 | 24 | 25 | 24 | 14 | 14 | 13 | 15 | 8 | 14 | 8 | 15 | 1 | 6 | 3 | 3 | 1 | 5 | 1 | 0 |
| 2020-02-01 | 1921 | 84 | 71 | 62 | 74 | 43 | 47 | 34 | 24 | 23 | 24 | 15 | 27 | 24 | 15 | 8 | 15 | 11 | 8 | 6 | 9 | 9 | 4 | 9 | 5 | 6 | 4 | 3 | 2 | 0 | 0 |
| 2020-02-02 | 2103 | 79 | 73 | 63 | 58 | 68 | 58 | 35 | 38 | 21 | 23 | 23 | 29 | 16 | 20 | 9 | 12 | 16 | 10 | 7 | 8 | 10 | 6 | 7 | 11 | 8 | 7 | 3 | 3 | 4 | 0 |
| 2020-02-03 | 2345 | 114 | 109 | 105 | 72 | 72 | 85 | 37 | 37 | 24 | 28 | 37 | 16 | 15 | 15 | 13 | 14 | 12 | 8 | 9 | 10 | 8 | 7 | 12 | 4 | 11 | 1 | 5 | 3 | 2 | 0 |
| 2020-02-04 | 3156 | 73 | 89 | 66 | 68 | 50 | 72 | 33 | 29 | 28 | 19 | 35 | 25 | 25 | 11 | 9 | 23 | 11 | 5 | 10 | 8 | 7 | 4 | 7 | 2 | 12 | 7 | 3 | 0 | 2 | 0 |
| 2020-02-05 | 2987 | 74 | 87 | 59 | 50 | 61 | 52 | 32 | 23 | 45 | 20 | 37 | 21 | 21 | 10 | 22 | 8 | 18 | 6 | 11 | 5 | 9 | 8 | 11 | 5 | 5 | 4 | 4 | 6 | 1 | 0 |
| 2020-02-06 | 2447 | 74 | 63 | 52 | 61 | 74 | 61 | 35 | 22 | 36 | 23 | 50 | 23 | 15 | 9 | 14 | 11 | 4 | 7 | 11 | 8 | 6 | 5 | 1 | 5 | 6 | 4 | 3 | 3 | 0 | 0 |
| 2020-02-07 | 2841 | 57 | 67 | 42 | 31 | 68 | 37 | 31 | 15 | 28 | 19 | 18 | 18 | 12 | 15 | 24 | 11 | 11 | 3 | 11 | 12 | 8 | 5 | 2 | 4 | 4 | 2 | 3 | 2 | 0 | 0 |
| 2020-02-08 | 2147 | 45 | 52 | 27 | 35 | 46 | 42 | 29 | 20 | 28 | 23 | 12 | 11 | 11 | 11 | 11 | 13 | 12 | 2 | 6 | 7 | 11 | 7 | 7 | 8 | 9 | 2 | 3 | 0 | 0 | 0 |
| 2020-02-09 | 2531 | 31 | 40 | 17 | 41 | 51 | 31 | 24 | 22 | 24 | 19 | 24 | 11 | 3 | 11 | 12 | 5 | 15 | 1 | 8 | 13 | 4 | 2 | 3 | 4 | 2 | 4 | 4 | 4 | 0 | 0 |
| 2020-02-10 | 2097 | 26 | 32 | 25 | 33 | 30 | 33 | 23 | 18 | 27 | 12 | 29 | 5 | 7 | 6 | 21 | 6 | 5 | 8 | 6 | 9 | 3 | 0 | 4 | 3 | 1 | 0 | 6 | 4 | 0 | 0 |
| 2020-02-11 | 1638 | 42 | 30 | 14 | 34 | 29 | 40 | 28 | 19 | 11 | 19 | 18 | 10 | 4 | 5 | 12 | 6 | 7 | 5 | 3 | 13 | 2 | 3 | 11 | 0 | 2 | 2 | 4 | 5 | 0 | 0 |

## Data 2: daily incoming immigration population size in each region¶

regions sorted by the mean value of "size"

(excluding Hubei)

In [7]:

```
df_curve_in = analyzer.df_curve_in
sorted_regions = df_curve_in.mean().sort_values(ascending=False).index
df_curve_in[sorted_regions]
```

Out[7]:

|  | Guangdong | Anhui | Henan | Jiangsu | Hunan | Sichuan | Hebei | Jiangxi | Guangxi | Beijing | Shandong | Shanghai | Chongqing | Hubei | Zhejiang | Guizhou | Shaanxi | Fujian | Yunnan | Liaoning | Shanxi | Tianjin | Neimenggu | Gansu | Heilongjiang | Jilin | Hainan | Ningxia | Xinjiang | Qinghai | Xizang |
| --- | --- | --- | --- | --- | --- | --- | --- | --- | --- | --- | --- | --- | --- | --- | --- | --- | --- | --- | --- | --- | --- | --- | --- | --- | --- | --- | --- | --- | --- | --- | --- |
| date |  |  |  |  |  |  |  |  |  |  |  |  |  |  |  |  |  |  |  |  |  |  |  |  |  |  |  |  |  |  |  |
| 2020-01-17 | 7.479702 | 19.064387 | 20.305274 | 12.567247 | 18.812995 | 13.799840 | 11.821172 | 16.720052 | 15.205417 | 7.910266 | 8.829421 | 6.046294 | 8.361760 | 12.762911 | 6.714187 | 8.927593 | 5.799600 | 4.881481 | 4.959209 | 3.440815 | 4.185173 | 2.927113 | 2.845368 | 3.189132 | 3.271331 | 2.588339 | 1.565503 | 0.885298 | 0.606820 | 0.534211 | 0.128012 |
| 2020-01-18 | 7.784878 | 22.622846 | 20.479068 | 13.981799 | 20.171819 | 15.186172 | 13.868982 | 18.286981 | 15.242159 | 7.295508 | 9.426974 | 5.728968 | 9.015300 | 13.375660 | 7.226885 | 9.102294 | 5.866085 | 5.531328 | 5.075881 | 3.554636 | 4.458532 | 3.224189 | 3.021527 | 3.447425 | 3.430156 | 2.666650 | 1.659496 | 0.947927 | 0.615794 | 0.500936 | 0.121014 |
| 2020-01-19 | 7.810182 | 23.948784 | 21.616405 | 13.344588 | 19.694178 | 15.184357 | 13.308008 | 18.350420 | 17.443674 | 7.676370 | 10.418998 | 5.616346 | 8.616035 | 13.805543 | 6.797326 | 8.307392 | 6.180073 | 5.599498 | 5.076432 | 3.711517 | 4.848206 | 2.832343 | 3.112733 | 3.597728 | 3.484134 | 2.745252 | 1.867309 | 0.918637 | 0.611032 | 0.487328 | 0.124740 |
| 2020-01-20 | 7.853468 | 24.634271 | 23.214892 | 13.721400 | 21.407749 | 15.983212 | 14.444114 | 16.788708 | 17.749109 | 7.020335 | 11.019694 | 5.336377 | 9.133074 | 14.511863 | 6.784430 | 8.469328 | 6.541398 | 5.630731 | 5.169647 | 3.919493 | 5.219251 | 2.769714 | 3.280435 | 3.882816 | 3.717641 | 2.970497 | 2.047648 | 0.952819 | 0.616864 | 0.473494 | 0.113854 |
| 2020-01-21 | 9.471946 | 28.747516 | 26.102736 | 15.827854 | 22.654534 | 18.145555 | 17.368571 | 17.720240 | 17.402947 | 7.075512 | 13.438937 | 5.228096 | 10.562724 | 16.044577 | 7.728113 | 8.914763 | 7.254036 | 6.170353 | 5.624024 | 4.796658 | 6.135361 | 3.017671 | 3.894901 | 4.654649 | 4.404229 | 3.528490 | 2.299784 | 1.040526 | 0.656100 | 0.514706 | 0.122310 |
| 2020-01-22 | 8.995601 | 24.880414 | 20.748409 | 14.501138 | 16.789972 | 15.364080 | 17.404956 | 14.143280 | 12.798583 | 5.902438 | 12.173846 | 4.306705 | 8.748518 | 12.086237 | 6.911762 | 6.798557 | 6.245456 | 5.429689 | 4.744591 | 4.589525 | 5.604649 | 2.747358 | 3.691624 | 4.277610 | 4.047894 | 3.404754 | 2.102274 | 0.936976 | 0.597488 | 0.470837 | 0.121792 |
| 2020-01-23 | 8.448980 | 21.878456 | 17.884897 | 13.420242 | 13.163893 | 14.352908 | 17.591256 | 11.299079 | 9.846652 | 4.839199 | 11.559154 | 3.733970 | 7.923517 | 8.887417 | 6.574252 | 5.730977 | 5.688436 | 4.815094 | 4.293130 | 4.231537 | 5.257742 | 2.418692 | 3.454326 | 4.154620 | 3.761802 | 3.296214 | 2.031869 | 0.828954 | 0.556600 | 0.443167 | 0.123703 |
| 2020-01-24 | 6.701584 | 17.378874 | 14.460638 | 11.781936 | 9.278388 | 11.957220 | 13.714337 | 8.061120 | 6.863519 | 3.716215 | 9.627984 | 2.867886 | 6.735247 | 5.143111 | 6.203077 | 4.335347 | 4.600217 | 4.018410 | 3.606120 | 3.585967 | 4.372672 | 1.860311 | 2.709482 | 3.389137 | 3.053117 | 2.695356 | 1.798589 | 0.740567 | 0.472003 | 0.423760 | 0.113627 |
| 2020-01-25 | 4.338328 | 8.598604 | 7.076678 | 6.485216 | 4.441262 | 7.145885 | 6.597482 | 3.643607 | 3.270035 | 2.549426 | 4.728683 | 1.950448 | 4.375361 | 1.977858 | 3.399602 | 2.789899 | 2.690431 | 1.872688 | 2.366042 | 1.961917 | 2.189884 | 1.258092 | 1.697598 | 1.913674 | 1.644138 | 1.590386 | 1.164326 | 0.515905 | 0.386208 | 0.343796 | 0.083106 |
| 2020-01-26 | 6.826939 | 6.463541 | 4.939250 | 7.244510 | 3.668846 | 6.573506 | 5.568620 | 2.697851 | 2.725261 | 5.077177 | 4.143150 | 3.631295 | 4.347756 | 1.118804 | 3.744112 | 3.235561 | 2.604960 | 1.620032 | 2.269231 | 2.303348 | 1.931364 | 2.219692 | 2.375471 | 1.627063 | 1.599296 | 1.965935 | 0.744973 | 0.533498 | 0.354521 | 0.376391 | 0.084758 |
| 2020-01-27 | 11.104646 | 4.522392 | 3.775378 | 8.798090 | 3.059143 | 5.987941 | 4.631677 | 2.093526 | 2.300594 | 6.344179 | 3.479728 | 5.219219 | 4.246344 | 0.821826 | 4.642272 | 3.330364 | 2.598091 | 1.667142 | 2.310606 | 2.771042 | 1.754460 | 2.512361 | 2.204917 | 1.500930 | 1.654603 | 2.024741 | 0.553716 | 0.584464 | 0.405778 | 0.420779 | 0.079510 |
| 2020-01-28 | 12.384122 | 3.153751 | 2.909099 | 8.506847 | 2.564006 | 4.885369 | 3.733873 | 1.695848 | 2.065824 | 6.012857 | 2.883114 | 5.859961 | 3.905982 | 0.688630 | 4.784735 | 2.944350 | 2.200284 | 1.631696 | 2.120677 | 2.689168 | 1.395760 | 2.104186 | 1.715256 | 1.215356 | 1.473455 | 1.665068 | 0.422140 | 0.608504 | 0.465296 | 0.434549 | 0.090461 |
| 2020-01-29 | 13.505130 | 2.359109 | 2.326320 | 6.328789 | 2.275517 | 4.057160 | 3.061541 | 1.455473 | 1.946203 | 5.713740 | 2.339474 | 5.270152 | 3.260153 | 0.594378 | 4.300776 | 2.470144 | 1.970276 | 1.645823 | 1.905703 | 2.290842 | 1.091750 | 1.900843 | 1.249636 | 0.924761 | 1.214482 | 1.324868 | 0.376034 | 0.511985 | 0.487426 | 0.407171 | 0.084046 |
| 2020-01-30 | 12.855251 | 2.006240 | 2.108981 | 5.440932 | 2.119964 | 3.531211 | 3.090604 | 1.340323 | 1.937682 | 6.108826 | 2.202455 | 5.109383 | 3.040189 | 0.532105 | 4.035679 | 2.249726 | 1.812812 | 1.809864 | 1.806203 | 2.293337 | 1.148062 | 2.178997 | 1.266548 | 0.819266 | 1.147802 | 1.217592 | 0.356594 | 0.438307 | 0.477479 | 0.359867 | 0.086508 |
| 2020-01-31 | 11.447471 | 1.695622 | 1.851854 | 4.709891 | 1.844370 | 2.921735 | 2.729538 | 1.128200 | 1.756080 | 5.842692 | 1.928286 | 4.885661 | 2.102533 | 0.463126 | 3.672637 | 1.901783 | 1.560060 | 1.773122 | 1.593983 | 1.911082 | 1.026302 | 1.896826 | 1.054199 | 0.748829 | 0.985284 | 0.961794 | 0.353095 | 0.400367 | 0.382223 | 0.345157 | 0.071183 |
| 2020-02-01 | 8.096501 | 1.480453 | 1.473520 | 3.659548 | 1.360444 | 2.343589 | 2.267579 | 0.805432 | 1.500379 | 5.547722 | 1.514635 | 4.136638 | 1.819454 | 0.395993 | 2.587302 | 1.439338 | 1.228414 | 1.260360 | 1.136333 | 1.536700 | 0.840942 | 1.636038 | 0.837054 | 0.580316 | 0.773744 | 0.787061 | 0.290272 | 0.280422 | 0.291211 | 0.262472 | 0.063115 |
| 2020-02-02 | 9.928300 | 1.690600 | 1.678061 | 4.213555 | 1.509095 | 2.562127 | 2.418174 | 0.895795 | 1.567901 | 6.069524 | 1.603476 | 4.972784 | 1.898154 | 0.516877 | 2.730834 | 1.620875 | 1.285049 | 1.465938 | 1.335496 | 1.537218 | 0.930625 | 1.600754 | 0.846029 | 0.648292 | 0.786413 | 0.829634 | 0.314215 | 0.274622 | 0.245754 | 0.255118 | 0.075881 |
| 2020-02-03 | 7.809631 | 1.350432 | 1.300244 | 3.826278 | 1.109700 | 2.129166 | 2.142094 | 0.642654 | 1.195916 | 5.613527 | 1.352506 | 4.566845 | 1.579468 | 0.399103 | 2.155864 | 1.093921 | 1.017068 | 1.111968 | 1.029316 | 1.163484 | 0.799956 | 1.409886 | 0.695434 | 0.576526 | 0.605232 | 0.567778 | 0.295067 | 0.262472 | 0.226152 | 0.247601 | 0.069854 |
| 2020-02-04 | 5.133553 | 1.090001 | 1.055786 | 2.660170 | 0.799405 | 1.548428 | 1.754816 | 0.451429 | 0.835855 | 3.436927 | 1.073444 | 3.224124 | 1.148969 | 0.371012 | 1.275523 | 0.812689 | 0.737197 | 0.708394 | 0.754337 | 0.827950 | 0.633485 | 0.952625 | 0.565412 | 0.431114 | 0.444463 | 0.412355 | 0.197510 | 0.184388 | 0.199843 | 0.176677 | 0.050414 |
| 2020-02-05 | 5.351670 | 1.024423 | 0.994615 | 2.472217 | 0.790657 | 1.403957 | 1.635390 | 0.420746 | 0.782298 | 2.883082 | 1.014509 | 3.362245 | 1.137694 | 0.375160 | 0.995846 | 0.775105 | 0.709268 | 0.646380 | 0.675929 | 0.741247 | 0.602867 | 0.859864 | 0.531976 | 0.391619 | 0.361876 | 0.356562 | 0.180922 | 0.162421 | 0.140681 | 0.142042 | 0.042736 |
| 2020-02-06 | 7.430130 | 0.942678 | 0.958003 | 2.514758 | 0.880373 | 1.365239 | 1.510682 | 0.444755 | 0.830444 | 2.490167 | 0.978253 | 3.721788 | 1.374538 | 0.407106 | 0.901724 | 0.846061 | 0.686329 | 0.685746 | 0.698900 | 0.708847 | 0.533920 | 0.726959 | 0.480719 | 0.375646 | 0.311980 | 0.333428 | 0.190382 | 0.151373 | 0.127526 | 0.138413 | 0.037746 |
| 2020-02-07 | 9.564966 | 0.975370 | 0.970866 | 2.572463 | 0.975953 | 1.347419 | 1.576487 | 0.449582 | 0.925960 | 2.587432 | 1.033916 | 3.959312 | 1.227668 | 0.385074 | 0.812009 | 0.845413 | 0.658757 | 0.742738 | 0.719539 | 0.707584 | 0.553878 | 0.787126 | 0.492707 | 0.351410 | 0.279904 | 0.309517 | 0.170780 | 0.141264 | 0.109026 | 0.134719 | 0.043157 |
| 2020-02-08 | 12.811284 | 0.982465 | 0.975856 | 3.114774 | 1.102151 | 1.319652 | 1.632830 | 0.470027 | 0.858244 | 2.889918 | 1.088089 | 4.873284 | 1.159402 | 0.393109 | 0.870232 | 0.776207 | 0.584302 | 0.860641 | 0.745265 | 0.692680 | 0.531911 | 0.756054 | 0.475567 | 0.333817 | 0.255377 | 0.291438 | 0.158695 | 0.135724 | 0.093895 | 0.141329 | 0.041440 |
| 2020-02-09 | 14.024016 | 1.034240 | 0.999151 | 2.872843 | 1.120489 | 1.312168 | 1.614719 | 0.442195 | 0.762955 | 4.055378 | 1.245715 | 5.491379 | 1.178485 | 0.409309 | 0.952398 | 0.731884 | 0.548759 | 0.891421 | 0.788260 | 0.760655 | 0.616442 | 0.982174 | 0.557960 | 0.329281 | 0.262051 | 0.308448 | 0.182023 | 0.147971 | 0.078246 | 0.140584 | 0.051160 |
| 2020-02-10 | 10.513962 | 0.991829 | 1.062428 | 2.365232 | 1.196694 | 1.270566 | 1.681139 | 0.446310 | 0.803326 | 4.224798 | 1.366470 | 4.554112 | 0.983470 | 0.457909 | 1.068358 | 0.759067 | 0.547884 | 0.840748 | 0.843242 | 0.835110 | 0.690282 | 1.027598 | 0.661057 | 0.348656 | 0.282074 | 0.336150 | 0.225536 | 0.157172 | 0.069530 | 0.168221 | 0.051224 |
| 2020-02-11 | 6.058314 | 0.864367 | 1.032847 | 1.703981 | 1.029024 | 1.042243 | 1.549141 | 0.370364 | 0.696697 | 2.585552 | 1.129302 | 2.694416 | 0.761854 | 0.366412 | 1.005048 | 0.683381 | 0.478451 | 0.623862 | 0.724594 | 0.682150 | 0.592078 | 0.740761 | 0.588449 | 0.323611 | 0.241931 | 0.297108 | 0.162745 | 0.142592 | 0.067230 | 0.143597 | 0.046559 |

## Data 3: daily immigration risk in each region¶

regions sorted by the mean value of "risk"

(excluding Hubei)

In [8]:

```
df_move_in_risk = analyzer.df_move_in_risk
sorted_regions = df_move_in_risk.mean().sort_values(ascending=False).index
df_move_in_risk[sorted_regions]
```

Out[8]:

|  | Guangdong | Hunan | Henan | Jiangsu | Shanghai | Chongqing | Anhui | Beijing | Sichuan | Jiangxi | Zhejiang | Guangxi | Hebei | Shaanxi | Shandong | Fujian | Guizhou | Yunnan | Tianjin | Hubei | Shanxi | Liaoning | Gansu | Hainan | Jilin | Neimenggu | Heilongjiang | Xinjiang | Ningxia | Qinghai | Xizang |
| --- | --- | --- | --- | --- | --- | --- | --- | --- | --- | --- | --- | --- | --- | --- | --- | --- | --- | --- | --- | --- | --- | --- | --- | --- | --- | --- | --- | --- | --- | --- | --- |
| date |  |  |  |  |  |  |  |  |  |  |  |  |  |  |  |  |  |  |  |  |  |  |  |  |  |  |  |  |  |  |  |
| 2020-01-17 | 5.912704 | 12.824819 | 15.050269 | 3.738756 | 1.572641 | 6.865841 | 5.477198 | 1.976775 | 3.612798 | 6.992326 | 2.910600 | 1.654349 | 2.089983 | 3.125404 | 2.521683 | 2.406570 | 1.623929 | 1.155000 | 0.308518 | 0.000000 | 1.052989 | 0.707776 | 0.731906 | 0.657355 | 0.365215 | 0.295065 | 0.500514 | 0.235203 | 0.115885 | 0.080826 | 0.026985 |
| 2020-01-18 | 26.683446 | 64.848363 | 69.727131 | 17.533176 | 20.638034 | 32.476717 | 26.821647 | 7.873312 | 17.196821 | 31.965643 | 13.786005 | 6.834584 | 10.224214 | 15.514621 | 11.678136 | 10.803790 | 7.194453 | 5.130701 | 1.543742 | 0.000000 | 4.947187 | 2.998691 | 3.275054 | 3.014308 | 1.519990 | 1.217071 | 2.346226 | 1.577173 | 0.641178 | 0.430204 | 0.110365 |
| 2020-01-19 | 57.596968 | 139.716407 | 147.892959 | 33.696419 | 12.327879 | 64.650417 | 52.577160 | 15.156225 | 42.305138 | 76.442346 | 27.304177 | 31.321861 | 20.556881 | 32.431788 | 23.784488 | 24.206068 | 16.507619 | 11.488981 | 3.038538 | 4.105768 | 10.670417 | 6.663658 | 7.136094 | 7.315371 | 3.414270 | 2.856244 | 5.395181 | 2.251102 | 1.354255 | 0.704141 | 0.174673 |
| 2020-01-20 | 86.908837 | 418.470118 | 293.289976 | 63.785300 | 20.349207 | 129.604713 | 104.515821 | 23.491444 | 115.937422 | 214.277638 | 50.595568 | 280.357823 | 66.604700 | 59.533263 | 49.854196 | 62.281518 | 56.894402 | 35.315925 | 7.919997 | 78.517885 | 24.593634 | 14.863893 | 14.648700 | 20.829286 | 8.105595 | 8.072167 | 11.965970 | 4.297935 | 2.273808 | 1.314797 | 0.369022 |
| 2020-01-21 | 144.275728 | 656.353944 | 491.639812 | 150.230072 | 33.679397 | 206.888906 | 238.495139 | 37.155636 | 214.719984 | 341.057923 | 89.840857 | 408.944895 | 138.556037 | 95.820012 | 101.738127 | 104.784938 | 98.570532 | 57.374047 | 14.438048 | 160.296557 | 46.561870 | 29.459155 | 26.628780 | 34.506655 | 16.355961 | 16.586826 | 23.347700 | 7.101233 | 3.750264 | 2.112870 | 0.519292 |
| 2020-01-22 | 152.845153 | 592.773305 | 495.436739 | 213.484309 | 41.936111 | 209.037099 | 294.146202 | 43.057102 | 212.204064 | 335.188674 | 102.668701 | 349.246459 | 188.561812 | 98.521450 | 125.372357 | 111.384644 | 90.763453 | 60.713687 | 20.251875 | 153.825162 | 56.896717 | 35.771674 | 29.995885 | 36.977318 | 20.761849 | 22.604550 | 28.806433 | 7.636858 | 4.352439 | 2.735279 | 0.895034 |
| 2020-01-23 | 235.210320 | 607.734825 | 618.334551 | 315.562228 | 85.104653 | 261.547379 | 500.651281 | 62.097572 | 342.035548 | 413.792604 | 138.758784 | 328.807268 | 294.479385 | 127.807771 | 188.848828 | 147.471872 | 137.872974 | 90.792822 | 30.273563 | 194.550006 | 84.940406 | 54.050694 | 47.681323 | 49.495308 | 33.501730 | 34.422359 | 44.746635 | 11.245483 | 6.597562 | 4.590680 | 1.849833 |
| 2020-01-24 | 308.513432 | 601.415832 | 606.471944 | 408.305348 | 111.166718 | 310.182380 | 555.618243 | 90.958455 | 457.338555 | 413.902237 | 200.936280 | 326.444054 | 310.411671 | 155.072388 | 237.115102 | 168.467821 | 162.559464 | 116.409520 | 38.845336 | 176.804219 | 97.789990 | 65.990044 | 56.819902 | 61.254539 | 46.867120 | 41.666691 | 55.714497 | 13.484707 | 10.002540 | 6.371776 | 2.717612 |
| 2020-01-25 | 605.991482 | 645.549925 | 542.152117 | 422.050101 | 157.995618 | 223.698197 | 481.943993 | 134.446806 | 426.988769 | 357.303014 | 241.880011 | 243.586200 | 241.194720 | 187.449337 | 214.186160 | 139.799875 | 169.868872 | 106.192242 | 48.303813 | 111.272511 | 88.331364 | 53.967634 | 58.119415 | 60.928618 | 41.248421 | 37.940976 | 45.023404 | 17.008446 | 12.126816 | 8.690314 | 3.025989 |
| 2020-01-26 | 1836.407731 | 683.501315 | 432.968763 | 684.906156 | 340.367072 | 378.215642 | 470.330534 | 301.676223 | 511.465470 | 331.877735 | 475.775493 | 267.006921 | 284.646157 | 224.847122 | 262.461095 | 196.604864 | 260.508622 | 145.748408 | 110.097369 | 78.363074 | 108.389113 | 71.929194 | 71.082649 | 53.404372 | 52.767066 | 58.890297 | 41.489427 | 21.651472 | 18.575294 | 14.438464 | 4.209475 |
| 2020-01-27 | 2167.384896 | 560.836700 | 422.396797 | 834.622048 | 471.933246 | 413.170970 | 405.269637 | 390.889623 | 448.915952 | 270.603937 | 506.800084 | 239.064657 | 266.140340 | 226.997826 | 250.282887 | 222.421417 | 254.048461 | 202.177101 | 134.370100 | 65.407406 | 101.235500 | 105.152192 | 90.442740 | 54.788925 | 64.667589 | 67.288120 | 50.649389 | 31.701700 | 27.878271 | 21.889250 | 4.426363 |
| 2020-01-28 | 1689.274792 | 501.020440 | 392.255900 | 849.314227 | 573.948040 | 348.056197 | 336.774268 | 371.253020 | 357.595346 | 248.798258 | 464.458992 | 225.438002 | 212.247915 | 184.943331 | 204.019526 | 203.814218 | 237.408240 | 147.341471 | 117.859854 | 63.560443 | 83.788565 | 103.492077 | 68.860392 | 41.954006 | 63.624928 | 61.565169 | 53.004295 | 37.208729 | 27.389574 | 22.849402 | 5.711822 |
| 2020-01-29 | 1750.275652 | 529.322981 | 397.880048 | 773.057171 | 640.516307 | 420.702180 | 312.561392 | 391.554032 | 315.066499 | 267.878750 | 477.385276 | 242.068949 | 179.313832 | 189.031273 | 194.375464 | 222.952373 | 230.864067 | 142.205478 | 119.939214 | 64.976987 | 77.406632 | 96.990814 | 55.747170 | 43.020704 | 55.508674 | 49.441833 | 49.859328 | 40.316970 | 23.835504 | 22.974246 | 5.592924 |
| 2020-01-30 | 1679.422820 | 537.233534 | 374.325533 | 726.003144 | 713.653043 | 390.833651 | 313.179945 | 435.651509 | 310.241976 | 268.942418 | 496.815920 | 282.466756 | 196.813655 | 190.025884 | 202.598091 | 273.509001 | 239.064701 | 151.858487 | 142.998652 | 70.491913 | 86.036310 | 99.530129 | 57.022170 | 47.294901 | 56.988785 | 53.517116 | 50.564369 | 42.912500 | 22.608762 | 20.788389 | 7.276119 |
| 2020-01-31 | 1512.507382 | 572.918682 | 385.688494 | 659.006508 | 691.732864 | 386.201919 | 308.651117 | 442.135200 | 303.292434 | 244.509457 | 486.009262 | 296.236999 | 188.134771 | 189.958990 | 189.315456 | 275.128133 | 217.605031 | 154.364323 | 128.686719 | 65.668015 | 86.201191 | 96.895850 | 59.882791 | 53.952841 | 54.665389 | 48.937279 | 49.971240 | 36.885188 | 23.855015 | 24.373517 | 6.260613 |
| 2020-02-01 | 1072.962050 | 590.195533 | 385.500577 | 488.790769 | 554.953927 | 298.719479 | 271.227317 | 386.732838 | 263.407005 | 202.958212 | 335.042155 | 269.605539 | 158.201036 | 168.595590 | 151.684960 | 200.352371 | 155.087043 | 101.409521 | 102.735333 | 58.337897 | 70.800673 | 79.797429 | 46.104397 | 45.314096 | 47.129201 | 39.228954 | 41.221465 | 27.901411 | 16.617219 | 17.092701 | 5.349468 |
| 2020-02-02 | 1426.539785 | 789.713685 | 559.140598 | 619.534611 | 695.544349 | 353.393566 | 328.723906 | 431.123174 | 407.499413 | 266.487427 | 395.994415 | 285.623548 | 194.383950 | 223.957277 | 179.873929 | 227.021316 | 191.970576 | 125.158107 | 102.730335 | 73.035007 | 85.075150 | 97.327728 | 57.682718 | 50.458813 | 71.626154 | 45.902308 | 47.869340 | 25.635607 | 16.889992 | 17.814836 | 5.494347 |
| 2020-02-03 | 1235.419617 | 706.517138 | 468.865010 | 596.050317 | 672.698066 | 306.250081 | 287.206367 | 1088.431972 | 237.602789 | 196.550199 | 317.251282 | 225.225022 | 174.860600 | 198.329965 | 158.689482 | 186.569994 | 117.547412 | 95.986047 | 90.111736 | 62.636932 | 73.165976 | 76.149911 | 49.713630 | 45.746153 | 40.337816 | 38.664648 | 36.109231 | 23.006466 | 18.198576 | 19.393184 | 5.311722 |
| 2020-02-04 | 838.324125 | 758.881352 | 524.353254 | 426.919446 | 499.554478 | 460.762685 | 268.958460 | 275.008136 | 200.426249 | 210.784347 | 194.579406 | 155.772148 | 145.124018 | 171.519397 | 141.833028 | 130.791844 | 84.353076 | 66.093331 | 231.437242 | 60.045203 | 57.796936 | 59.282433 | 39.864847 | 32.098817 | 33.453520 | 35.478045 | 27.027363 | 21.759607 | 9.084577 | 13.610999 | 3.779724 |
| 2020-02-05 | 877.419676 | 834.424662 | 569.458280 | 398.548607 | 505.798012 | 470.462243 | 286.932845 | 254.294717 | 196.187379 | 190.986679 | 149.418387 | 140.535064 | 132.578287 | 182.088561 | 132.571850 | 116.014739 | 82.044110 | 54.413282 | 230.000529 | 58.130755 | 57.868340 | 58.839980 | 34.984952 | 28.776539 | 32.919515 | 31.462527 | 24.410644 | 16.775679 | 8.959365 | 10.101928 | 3.280128 |
| 2020-02-06 | 1133.711526 | 849.987261 | 551.736843 | 375.884710 | 538.474987 | 498.064524 | 247.580048 | 217.591771 | 197.202192 | 199.744540 | 137.920821 | 132.765555 | 123.945297 | 159.488150 | 122.952438 | 106.698649 | 77.331940 | 52.076047 | 53.654575 | 54.448107 | 45.584721 | 56.245466 | 32.644805 | 28.111637 | 33.125645 | 27.685028 | 20.152915 | 15.156627 | 8.152727 | 8.935183 | 2.488024 |
| 2020-02-07 | 1161.420258 | 825.818955 | 536.245311 | 387.692010 | 546.327305 | 450.831142 | 235.200333 | 211.451896 | 193.472227 | 188.407665 | 120.505029 | 118.071794 | 118.910773 | 154.888085 | 118.691535 | 102.841825 | 67.904011 | 50.986476 | 60.647240 | 46.121006 | 45.642981 | 49.114015 | 25.352363 | 22.344293 | 26.419800 | 27.905632 | 18.332902 | 10.987597 | 7.055007 | 9.128762 | 2.913162 |
| 2020-02-08 | 1416.788367 | 766.998535 | 474.229813 | 410.875756 | 619.806189 | 417.669325 | 215.010937 | 202.319113 | 176.260243 | 187.166928 | 114.189354 | 103.636176 | 109.830541 | 128.987558 | 106.125366 | 109.990290 | 59.336585 | 53.412979 | 51.877099 | 43.103873 | 40.169425 | 43.825007 | 24.752045 | 19.130024 | 21.192526 | 22.259208 | 13.103052 | 9.507114 | 5.231073 | 9.232968 | 2.801408 |
| 2020-02-09 | 1389.256890 | 765.168629 | 515.217512 | 334.415611 | 606.842936 | 450.869700 | 206.465514 | 253.583217 | 166.956794 | 161.817840 | 110.678933 | 80.129370 | 95.218837 | 143.156245 | 114.029281 | 98.071308 | 50.656079 | 51.089627 | 58.575164 | 37.933098 | 39.459526 | 35.967866 | 19.400293 | 18.281172 | 17.376634 | 23.909273 | 13.627580 | 8.021905 | 3.973238 | 6.635124 | 3.447205 |
| 2020-02-10 | 1033.524567 | 722.555460 | 463.851777 | 227.648178 | 426.236155 | 382.084430 | 166.711445 | 240.253278 | 164.519874 | 159.130001 | 106.401473 | 73.255502 | 85.262653 | 118.815330 | 101.228098 | 79.898935 | 48.267337 | 51.769181 | 60.620599 | 38.354154 | 43.247134 | 40.273597 | 18.546359 | 17.967876 | 18.298695 | 24.522247 | 12.185219 | 5.664384 | 5.058389 | 8.849928 | 2.752733 |
| 2020-02-11 | 622.657155 | 633.647151 | 431.410050 | 149.192380 | 241.783186 | 316.878073 | 151.623750 | 146.647102 | 127.217456 | 126.630329 | 92.515271 | 63.989130 | 66.180706 | 111.741371 | 79.783266 | 60.787366 | 39.923311 | 40.164078 | 37.369551 | 29.179884 | 29.104640 | 29.031127 | 15.754008 | 12.513365 | 15.886632 | 22.397421 | 8.094400 | 5.543308 | 3.878699 | 6.516279 | 1.962831 |

## model output result¶

The NaN values indicate that there were no diagnosed people in the regions on that day. The calculation results are available from the first day when the number of newly diagnosed people is not zero.

The regions are sorted by the values of "offset + window" in 14 Feb.

In [9]:

```
df_move_inc_corr = analyzer.df_move_inc_corr
s_offset = df_move_inc_corr.loc['2020-02-11'][2::4]
s_window = df_move_inc_corr.loc['2020-02-11'][3::4]
sorted_regions = df_move_inc_corr.columns.levels[0].values[np.argsort(s_offset.values + s_window.values)]
df_move_inc_corr = df_move_inc_corr[sorted_regions]
del df_move_inc_corr['Hubei']
df_move_inc_corr
```

Out[9]:

|  | Shanghai | | | | Qinghai | | | | Liaoning | | | | Xizang | | | | Fujian | | | | Yunnan | | | | Ningxia | | | | Zhejiang | | | | Chongqing | | | | Beijing | | | | Guangdong | | | | Shaanxi | | | | Jilin | | | | Tianjin | | | | Hunan | | | | Gansu | | | | Sichuan | | | | Jiangsu | | | | Shandong | | | | Shanxi | | | | Neimenggu | | | | Guangxi | | | | Hainan | | | | Hebei | | | | Anhui | | | | Henan | | | | Jiangxi | | | | Heilongjiang | | | | Xinjiang | | | | Guizhou | | | |
| --- | --- | --- | --- | --- | --- | --- | --- | --- | --- | --- | --- | --- | --- | --- | --- | --- | --- | --- | --- | --- | --- | --- | --- | --- | --- | --- | --- | --- | --- | --- | --- | --- | --- | --- | --- | --- | --- | --- | --- | --- | --- | --- | --- | --- | --- | --- | --- | --- | --- | --- | --- | --- | --- | --- | --- | --- | --- | --- | --- | --- | --- | --- | --- | --- | --- | --- | --- | --- | --- | --- | --- | --- | --- | --- | --- | --- | --- | --- | --- | --- | --- | --- | --- | --- | --- | --- | --- | --- | --- | --- | --- | --- | --- | --- | --- | --- | --- | --- | --- | --- | --- | --- | --- | --- | --- | --- | --- | --- | --- | --- | --- | --- | --- | --- | --- | --- | --- | --- | --- | --- |
|  | corr | corr delta | offset | window | corr | corr delta | offset | window | corr | corr delta | offset | window | corr | corr delta | offset | window | corr | corr delta | offset | window | corr | corr delta | offset | window | corr | corr delta | offset | window | corr | corr delta | offset | window | corr | corr delta | offset | window | corr | corr delta | offset | window | corr | corr delta | offset | window | corr | corr delta | offset | window | corr | corr delta | offset | window | corr | corr delta | offset | window | corr | corr delta | offset | window | corr | corr delta | offset | window | corr | corr delta | offset | window | corr | corr delta | offset | window | corr | corr delta | offset | window | corr | corr delta | offset | window | corr | corr delta | offset | window | corr | corr delta | offset | window | corr | corr delta | offset | window | corr | corr delta | offset | window | corr | corr delta | offset | window | corr | corr delta | offset | window | corr | corr delta | offset | window | corr | corr delta | offset | window | corr | corr delta | offset | window | corr | corr delta | offset | window |
| 2020-01-17 | NaN | NaN | NaN | NaN | NaN | NaN | NaN | NaN | NaN | NaN | NaN | NaN | NaN | NaN | NaN | NaN | NaN | NaN | NaN | NaN | NaN | NaN | NaN | NaN | NaN | NaN | NaN | NaN | NaN | NaN | NaN | NaN | NaN | NaN | NaN | NaN | NaN | NaN | NaN | NaN | NaN | NaN | NaN | NaN | NaN | NaN | NaN | NaN | NaN | NaN | NaN | NaN | NaN | NaN | NaN | NaN | NaN | NaN | NaN | NaN | NaN | NaN | NaN | NaN | NaN | NaN | NaN | NaN | NaN | NaN | NaN | NaN | NaN | NaN | NaN | NaN | NaN | NaN | NaN | NaN | NaN | NaN | NaN | NaN | NaN | NaN | NaN | NaN | NaN | NaN | NaN | NaN | NaN | NaN | NaN | NaN | NaN | NaN | NaN | NaN | NaN | NaN | NaN | NaN | NaN | NaN | NaN | NaN | NaN | NaN | NaN | NaN | NaN | NaN | NaN | NaN | NaN | NaN | NaN | NaN |
| 2020-01-18 | NaN | NaN | NaN | NaN | NaN | NaN | NaN | NaN | NaN | NaN | NaN | NaN | NaN | NaN | NaN | NaN | NaN | NaN | NaN | NaN | NaN | NaN | NaN | NaN | NaN | NaN | NaN | NaN | NaN | NaN | NaN | NaN | NaN | NaN | NaN | NaN | NaN | NaN | NaN | NaN | NaN | NaN | NaN | NaN | NaN | NaN | NaN | NaN | NaN | NaN | NaN | NaN | NaN | NaN | NaN | NaN | NaN | NaN | NaN | NaN | NaN | NaN | NaN | NaN | NaN | NaN | NaN | NaN | NaN | NaN | NaN | NaN | NaN | NaN | NaN | NaN | NaN | NaN | NaN | NaN | NaN | NaN | NaN | NaN | NaN | NaN | NaN | NaN | NaN | NaN | NaN | NaN | NaN | NaN | NaN | NaN | NaN | NaN | NaN | NaN | NaN | NaN | NaN | NaN | NaN | NaN | NaN | NaN | NaN | NaN | NaN | NaN | NaN | NaN | NaN | NaN | NaN | NaN | NaN | NaN |
| 2020-01-19 | NaN | NaN | NaN | NaN | NaN | NaN | NaN | NaN | NaN | NaN | NaN | NaN | NaN | NaN | NaN | NaN | NaN | NaN | NaN | NaN | NaN | NaN | NaN | NaN | NaN | NaN | NaN | NaN | NaN | NaN | NaN | NaN | NaN | NaN | NaN | NaN | NaN | NaN | NaN | NaN | 1.000000 | 0.083189 | 2.0 | 5.0 | NaN | NaN | NaN | NaN | NaN | NaN | NaN | NaN | NaN | NaN | NaN | NaN | NaN | NaN | NaN | NaN | NaN | NaN | NaN | NaN | NaN | NaN | NaN | NaN | NaN | NaN | NaN | NaN | NaN | NaN | NaN | NaN | NaN | NaN | NaN | NaN | NaN | NaN | NaN | NaN | NaN | NaN | NaN | NaN | NaN | NaN | NaN | NaN | NaN | NaN | NaN | NaN | NaN | NaN | NaN | NaN | NaN | NaN | NaN | NaN | NaN | NaN | NaN | NaN | NaN | NaN | NaN | NaN | NaN | NaN | NaN | NaN | NaN | NaN | NaN | NaN |
| 2020-01-20 | 1.000000 | 0.507367 | 3.0 | 1.0 | NaN | NaN | NaN | NaN | NaN | NaN | NaN | NaN | NaN | NaN | NaN | NaN | NaN | NaN | NaN | NaN | NaN | NaN | NaN | NaN | NaN | NaN | NaN | NaN | NaN | NaN | NaN | NaN | NaN | NaN | NaN | NaN | 1.000000 | 0.185126 | 3.0 | 3.0 | 0.998194 | 0.165575 | 3.0 | 1.0 | NaN | NaN | NaN | NaN | NaN | NaN | NaN | NaN | NaN | NaN | NaN | NaN | NaN | NaN | NaN | NaN | NaN | NaN | NaN | NaN | NaN | NaN | NaN | NaN | NaN | NaN | NaN | NaN | NaN | NaN | NaN | NaN | NaN | NaN | NaN | NaN | NaN | NaN | NaN | NaN | NaN | NaN | NaN | NaN | NaN | NaN | NaN | NaN | NaN | NaN | NaN | NaN | NaN | NaN | NaN | NaN | NaN | NaN | NaN | NaN | NaN | NaN | NaN | NaN | NaN | NaN | NaN | NaN | NaN | NaN | NaN | NaN | NaN | NaN | NaN | NaN |
| 2020-01-21 | 0.998789 | 0.210157 | 3.0 | 1.0 | NaN | NaN | NaN | NaN | NaN | NaN | NaN | NaN | NaN | NaN | NaN | NaN | NaN | NaN | NaN | NaN | NaN | NaN | NaN | NaN | NaN | NaN | NaN | NaN | 1.000000 | 0.142957 | 4.0 | 1.0 | 1.000000 | 0.177534 | 4.0 | 1.0 | 0.911712 | 0.038393 | 1.0 | 2.0 | 0.777537 | 0.081624 | 1.0 | 1.0 | NaN | NaN | NaN | NaN | NaN | NaN | NaN | NaN | 1.000000 | 0.133157 | 4.0 | 1.0 | NaN | NaN | NaN | NaN | NaN | NaN | NaN | NaN | NaN | NaN | NaN | NaN | NaN | NaN | NaN | NaN | NaN | NaN | NaN | NaN | NaN | NaN | NaN | NaN | NaN | NaN | NaN | NaN | NaN | NaN | NaN | NaN | NaN | NaN | NaN | NaN | NaN | NaN | NaN | NaN | NaN | NaN | NaN | NaN | 1.000000 | 0.161499 | 4.0 | 1.0 | 1.000000 | 0.177849 | 4.0 | 3.0 | NaN | NaN | NaN | NaN | NaN | NaN | NaN | NaN | NaN | NaN | NaN | NaN |
| 2020-01-22 | 0.975092 | 0.114766 | 3.0 | 1.0 | NaN | NaN | NaN | NaN | 1.000000 | 0.303159 | 5.0 | 1.0 | NaN | NaN | NaN | NaN | 1.000000 | 0.395884 | 5.0 | 3.0 | NaN | NaN | NaN | NaN | NaN | NaN | NaN | NaN | 0.918001 | 0.002050 | 3.0 | 1.0 | 0.881693 | 0.007938 | 0.0 | 2.0 | 0.834677 | 0.000000 | 0.0 | 1.0 | 0.587917 | 0.000000 | 0.0 | 1.0 | NaN | NaN | NaN | NaN | NaN | NaN | NaN | NaN | 0.919850 | 0.002633 | 3.0 | 1.0 | NaN | NaN | NaN | NaN | NaN | NaN | NaN | NaN | 1.000000 | 0.428725 | 5.0 | 1.0 | 1.000000 | 0.219400 | 5.0 | 1.0 | NaN | NaN | NaN | NaN | 1.000000 | 0.303676 | 5.0 | 1.0 | NaN | NaN | NaN | NaN | 1.000000 | 0.556131 | 5.0 | 7.0 | 1.000000 | 0.394112 | 5.0 | 1.0 | 1.000000 | 0.249438 | 5.0 | 5.0 | 1.000000 | 0.285206 | 5.0 | 3.0 | 0.997817 | 0.232285 | 3.0 | 5.0 | 0.818390 | 0.000000 | 0.0 | 1.0 | NaN | NaN | NaN | NaN | NaN | NaN | NaN | NaN | NaN | NaN | NaN | NaN |
| 2020-01-23 | 0.875797 | 0.337711 | 3.0 | 1.0 | NaN | NaN | NaN | NaN | 0.819636 | 0.139738 | 1.0 | 1.0 | NaN | NaN | NaN | NaN | 0.993630 | 0.212661 | 4.0 | 1.0 | NaN | NaN | NaN | NaN | 1.000000 | 0.260030 | 6.0 | 2.0 | 0.987268 | 0.191815 | 5.0 | 2.0 | 0.964042 | 0.178987 | 3.0 | 5.0 | 0.920736 | 0.001961 | 2.0 | 1.0 | 0.802286 | 0.000000 | 0.0 | 1.0 | 1.000000 | 0.382347 | 6.0 | 1.0 | 1.000000 | 0.222577 | 6.0 | 1.0 | 0.694104 | 0.000832 | 0.0 | 2.0 | 1.000000 | 0.600504 | 6.0 | 3.0 | 1.000000 | 0.246852 | 6.0 | 1.0 | 0.947776 | 0.158645 | 1.0 | 4.0 | 0.998676 | 0.182132 | 5.0 | 9.0 | 1.000000 | 0.254396 | 6.0 | 1.0 | 0.497612 | 0.156845 | 1.0 | 1.0 | 1.000000 | 0.239566 | 6.0 | 3.0 | 0.999912 | 0.615204 | 5.0 | 3.0 | 0.935402 | 0.166302 | 2.0 | 1.0 | 0.935043 | 0.072245 | 1.0 | 1.0 | 0.888869 | 0.143565 | 1.0 | 1.0 | 0.994432 | 0.161156 | 3.0 | 2.0 | 0.924100 | 0.099303 | 3.0 | 1.0 | 1.000000 | 0.242519 | 6.0 | 7.0 | 1.000000 | 0.289167 | 6.0 | 1.0 | 1.000000 | 0.334092 | 6.0 | 1.0 |
| 2020-01-24 | 0.940807 | 0.146372 | 3.0 | 1.0 | NaN | NaN | NaN | NaN | 0.972306 | 0.213134 | 7.0 | 3.0 | NaN | NaN | NaN | NaN | 0.996053 | 0.342147 | 6.0 | 2.0 | 1.000000 | 0.333347 | 7.0 | 5.0 | 0.795900 | 0.090140 | 2.0 | 1.0 | 0.887503 | 0.107074 | 2.0 | 1.0 | 0.988956 | 0.156934 | 3.0 | 4.0 | 0.928124 | 0.034250 | 2.0 | 1.0 | 0.884850 | 0.000000 | 0.0 | 1.0 | 0.849901 | 0.132339 | 2.0 | 1.0 | 0.735244 | 0.080261 | 2.0 | 1.0 | 0.844489 | 0.008466 | 1.0 | 1.0 | 0.907053 | 0.415253 | 2.0 | 2.0 | 0.923515 | 0.065210 | 2.0 | 4.0 | 0.977393 | 0.080121 | 1.0 | 4.0 | 0.969064 | 0.063354 | 2.0 | 1.0 | 0.970932 | 0.100528 | 5.0 | 1.0 | 0.979864 | 0.316332 | 7.0 | 9.0 | 0.934167 | 0.070523 | 2.0 | 1.0 | 0.976587 | 0.524882 | 2.0 | 2.0 | 0.966278 | 0.117130 | 2.0 | 9.0 | 0.989196 | 0.265188 | 4.0 | 1.0 | 0.960269 | 0.149399 | 3.0 | 2.0 | 0.991417 | 0.390477 | 6.0 | 7.0 | 0.985930 | 0.304338 | 4.0 | 6.0 | 0.952058 | 0.076580 | 2.0 | 5.0 | 0.837296 | 0.106317 | 5.0 | 1.0 | 0.799186 | 0.163603 | 2.0 | 1.0 |
| 2020-01-25 | 0.833522 | 0.156431 | 3.0 | 1.0 | 1.000000 | 0.280965 | 8.0 | 1.0 | 0.930973 | 0.135635 | 3.0 | 1.0 | NaN | NaN | NaN | NaN | 0.924437 | 0.227524 | 3.0 | 1.0 | 0.967649 | 0.234236 | 4.0 | 1.0 | 0.735592 | 0.041854 | 2.0 | 1.0 | 0.939034 | 0.060036 | 2.0 | 1.0 | 0.947887 | 0.108869 | 3.0 | 1.0 | 0.956258 | 0.026480 | 2.0 | 1.0 | 0.917913 | 0.015115 | 0.0 | 4.0 | 0.981689 | 0.282465 | 7.0 | 7.0 | 0.493663 | 0.000000 | 0.0 | 1.0 | 0.796487 | 0.000000 | 0.0 | 1.0 | 0.932272 | 0.366489 | 2.0 | 3.0 | 0.959748 | 0.073444 | 2.0 | 2.0 | 0.985815 | 0.076350 | 1.0 | 3.0 | 0.982891 | 0.060704 | 2.0 | 1.0 | 0.980191 | 0.127087 | 2.0 | 4.0 | 0.854716 | 0.132905 | 3.0 | 1.0 | 0.981591 | 0.249204 | 5.0 | 1.0 | 0.981045 | 0.556924 | 2.0 | 2.0 | 0.739979 | 0.000000 | 0.0 | 1.0 | 0.942078 | 0.201475 | 3.0 | 1.0 | 0.960520 | 0.121957 | 1.0 | 1.0 | 0.998072 | 0.497758 | 6.0 | 1.0 | 0.995238 | 0.411688 | 4.0 | 1.0 | 0.964908 | 0.099452 | 3.0 | 1.0 | 0.759965 | 0.037893 | 2.0 | 1.0 | 0.699622 | 0.074122 | 2.0 | 1.0 |
| 2020-01-26 | 0.832341 | 0.092453 | 3.0 | 2.0 | 0.994995 | 0.103866 | 7.0 | 5.0 | 0.850410 | 0.121755 | 1.0 | 1.0 | NaN | NaN | NaN | NaN | 0.955699 | 0.192704 | 3.0 | 7.0 | 0.978369 | 0.147280 | 4.0 | 1.0 | 0.886726 | 0.019492 | 2.0 | 1.0 | 0.825240 | 0.146591 | 1.0 | 2.0 | 0.965610 | 0.072171 | 3.0 | 1.0 | 0.952460 | 0.101686 | 2.0 | 1.0 | 0.931411 | 0.181651 | 2.0 | 1.0 | 0.917898 | 0.124981 | 4.0 | 1.0 | 0.616116 | 0.000000 | 0.0 | 1.0 | 0.876317 | 0.018454 | 0.0 | 2.0 | 0.952758 | 0.330229 | 2.0 | 4.0 | 0.978747 | 0.135542 | 5.0 | 1.0 | 0.990368 | 0.082588 | 2.0 | 2.0 | 0.988095 | 0.035383 | 2.0 | 1.0 | 0.989277 | 0.109506 | 2.0 | 3.0 | 0.866993 | 0.079887 | 3.0 | 1.0 | 0.995592 | 0.165244 | 5.0 | 1.0 | 0.965653 | 0.555356 | 2.0 | 2.0 | 0.730620 | 0.000000 | 0.0 | 1.0 | 0.936889 | 0.157071 | 1.0 | 3.0 | 0.918533 | 0.092147 | 1.0 | 1.0 | 0.983495 | 0.519172 | 4.0 | 1.0 | 0.972005 | 0.374167 | 4.0 | 1.0 | 0.960015 | 0.102297 | 0.0 | 5.0 | 0.729420 | 0.034282 | 2.0 | 1.0 | 0.763695 | 0.056000 | 2.0 | 1.0 |
| 2020-01-27 | 0.830059 | 0.071190 | 6.0 | 1.0 | 0.905532 | 0.047131 | 5.0 | 1.0 | 0.841992 | 0.135491 | 1.0 | 1.0 | NaN | NaN | NaN | NaN | 0.757819 | 0.145357 | 2.0 | 1.0 | 0.949356 | 0.091669 | 4.0 | 2.0 | 0.940315 | 0.005071 | 2.0 | 2.0 | 0.877974 | 0.091540 | 2.0 | 1.0 | 0.936981 | 0.065773 | 2.0 | 2.0 | 0.826853 | 0.090410 | 4.0 | 2.0 | 0.935458 | 0.107498 | 3.0 | 2.0 | 0.897052 | 0.075636 | 4.0 | 1.0 | 0.685812 | 0.000000 | 0.0 | 1.0 | 0.967385 | 0.047196 | 1.0 | 1.0 | 0.967159 | 0.397268 | 2.0 | 10.0 | 0.952753 | 0.079498 | 5.0 | 1.0 | 0.986027 | 0.071812 | 2.0 | 1.0 | 0.990198 | 0.017393 | 1.0 | 4.0 | 0.991105 | 0.099118 | 2.0 | 2.0 | 0.920604 | 0.139990 | 3.0 | 1.0 | 0.874382 | 0.120623 | 2.0 | 1.0 | 0.944423 | 0.533732 | 2.0 | 2.0 | 0.780148 | 0.077637 | 3.0 | 1.0 | 0.937737 | 0.321739 | 4.0 | 1.0 | 0.863949 | 0.159009 | 0.0 | 10.0 | 0.963529 | 0.510076 | 4.0 | 1.0 | 0.973337 | 0.512760 | 4.0 | 1.0 | 0.970917 | 0.119458 | 0.0 | 10.0 | 0.903131 | 0.044868 | 9.0 | 5.0 | 0.797751 | 0.045155 | 2.0 | 1.0 |
| 2020-01-28 | 0.841183 | 0.052568 | 6.0 | 1.0 | 0.583131 | 0.000000 | 0.0 | 1.0 | 0.887108 | 0.103997 | 1.0 | 1.0 | NaN | NaN | NaN | NaN | 0.820783 | 0.146107 | 2.0 | 1.0 | 0.951086 | 0.304539 | 8.0 | 1.0 | 0.774514 | 0.000000 | 0.0 | 1.0 | 0.959823 | 0.226448 | 2.0 | 1.0 | 0.895438 | 0.047403 | 3.0 | 1.0 | 0.887173 | 0.069557 | 4.0 | 2.0 | 0.953128 | 0.096325 | 4.0 | 1.0 | 0.856229 | 0.022027 | 4.0 | 1.0 | 0.645440 | 0.000000 | 0.0 | 1.0 | 0.744750 | 0.000000 | 0.0 | 1.0 | 0.947422 | 0.544331 | 3.0 | 9.0 | 0.944360 | 0.058970 | 2.0 | 2.0 | 0.973860 | 0.065290 | 0.0 | 4.0 | 0.994293 | 0.024088 | 1.0 | 4.0 | 0.989329 | 0.175364 | 2.0 | 4.0 | 0.942834 | 0.190457 | 3.0 | 2.0 | 0.833562 | 0.058916 | 2.0 | 2.0 | 0.945341 | 0.538410 | 2.0 | 2.0 | 0.831722 | 0.174733 | 3.0 | 1.0 | 0.960389 | 0.408718 | 4.0 | 1.0 | 0.912882 | 0.339800 | 4.0 | 1.0 | 0.956703 | 0.519478 | 4.0 | 1.0 | 0.963264 | 0.659665 | 4.0 | 8.0 | 0.965332 | 0.095664 | 0.0 | 5.0 | 0.855950 | 0.000000 | 0.0 | 1.0 | 0.595745 | 0.000000 | 0.0 | 1.0 |
| 2020-01-29 | 0.873984 | 0.012525 | 6.0 | 1.0 | 0.434434 | 0.000000 | 0.0 | 1.0 | 0.770369 | 0.045117 | 1.0 | 1.0 | 0.908678 | 0.443191 | 10.0 | 3.0 | 0.838905 | 0.123689 | 2.0 | 1.0 | 0.937027 | 0.265698 | 4.0 | 5.0 | 0.840575 | 0.048959 | 3.0 | 1.0 | 0.975930 | 0.207245 | 2.0 | 1.0 | 0.822678 | 0.000000 | 0.0 | 1.0 | 0.779336 | 0.000000 | 0.0 | 1.0 | 0.963666 | 0.108256 | 5.0 | 1.0 | 0.829687 | 0.000000 | 0.0 | 1.0 | 0.761749 | 0.134225 | 9.0 | 1.0 | 0.721044 | 0.000000 | 0.0 | 1.0 | 0.948791 | 0.547480 | 2.0 | 9.0 | 0.874848 | 0.000000 | 0.0 | 1.0 | 0.949656 | 0.184419 | 0.0 | 10.0 | 0.994239 | 0.032065 | 0.0 | 5.0 | 0.979380 | 0.166382 | 2.0 | 1.0 | 0.955549 | 0.246363 | 3.0 | 2.0 | 0.773470 | 0.000000 | 0.0 | 1.0 | 0.818108 | 0.487348 | 1.0 | 10.0 | 0.768622 | 0.119248 | 3.0 | 1.0 | 0.968887 | 0.497757 | 3.0 | 5.0 | 0.932344 | 0.434067 | 4.0 | 3.0 | 0.930403 | 0.566056 | 2.0 | 10.0 | 0.970429 | 0.741688 | 5.0 | 8.0 | 0.961637 | 0.084017 | 3.0 | 1.0 | 0.705177 | 0.000000 | 0.0 | 1.0 | 0.675835 | 0.031230 | 3.0 | 1.0 |
| 2020-01-30 | 0.922925 | 0.021221 | 6.0 | 1.0 | 0.515695 | 0.000000 | 0.0 | 1.0 | 0.782095 | 0.039229 | 1.0 | 1.0 | 0.551845 | 0.204607 | 5.0 | 1.0 | 0.854433 | 0.111080 | 2.0 | 1.0 | 0.862949 | 0.211868 | 1.0 | 2.0 | 0.841536 | 0.028652 | 0.0 | 4.0 | 0.979928 | 0.176315 | 2.0 | 1.0 | 0.866391 | 0.045294 | 3.0 | 1.0 | 0.807751 | 0.000000 | 0.0 | 1.0 | 0.963117 | 0.123176 | 6.0 | 1.0 | 0.843102 | 0.085677 | 4.0 | 1.0 | 0.552406 | 0.014489 | 2.0 | 1.0 | 0.724625 | 0.000000 | 0.0 | 1.0 | 0.953622 | 0.548069 | 2.0 | 7.0 | 0.876466 | 0.000000 | 0.0 | 1.0 | 0.959104 | 0.267647 | 0.0 | 10.0 | 0.993912 | 0.073217 | 1.0 | 4.0 | 0.979198 | 0.185917 | 2.0 | 3.0 | 0.919011 | 0.205345 | 1.0 | 5.0 | 0.768994 | 0.000000 | 0.0 | 1.0 | 0.801976 | 0.461321 | 3.0 | 1.0 | 0.719459 | 0.082685 | 1.0 | 3.0 | 0.975244 | 0.527638 | 4.0 | 3.0 | 0.935533 | 0.453159 | 4.0 | 2.0 | 0.946263 | 0.635279 | 2.0 | 10.0 | 0.980273 | 0.804447 | 6.0 | 10.0 | 0.903653 | 0.138722 | 0.0 | 10.0 | 0.743677 | 0.000000 | 0.0 | 1.0 | 0.745204 | 0.061911 | 3.0 | 1.0 |
| 2020-01-31 | 0.930131 | 0.009771 | 0.0 | 4.0 | 0.514544 | 0.000000 | 0.0 | 1.0 | 0.820739 | 0.102337 | 4.0 | 1.0 | 0.371958 | 0.076279 | 3.0 | 1.0 | 0.875006 | 0.092787 | 2.0 | 1.0 | 0.874948 | 0.196758 | 1.0 | 2.0 | 0.875843 | 0.049526 | 0.0 | 7.0 | 0.940650 | 0.142932 | 2.0 | 1.0 | 0.880132 | 0.045675 | 3.0 | 1.0 | 0.841326 | 0.000000 | 0.0 | 1.0 | 0.977417 | 0.238320 | 6.0 | 2.0 | 0.840428 | 0.070809 | 4.0 | 1.0 | 0.604526 | 0.034325 | 5.0 | 1.0 | 0.633889 | 0.000000 | 0.0 | 1.0 | 0.958522 | 0.537796 | 2.0 | 7.0 | 0.855363 | 0.000000 | 0.0 | 1.0 | 0.963080 | 0.297051 | 0.0 | 10.0 | 0.992935 | 0.083248 | 0.0 | 5.0 | 0.978527 | 0.184296 | 2.0 | 3.0 | 0.922901 | 0.214044 | 1.0 | 5.0 | 0.777553 | 0.000000 | 0.0 | 1.0 | 0.815438 | 0.453595 | 0.0 | 10.0 | 0.726540 | 0.068952 | 0.0 | 5.0 | 0.975224 | 0.536611 | 4.0 | 3.0 | 0.938810 | 0.519186 | 1.0 | 10.0 | 0.954776 | 0.665304 | 2.0 | 10.0 | 0.951364 | 0.791219 | 6.0 | 4.0 | 0.913705 | 0.225638 | 2.0 | 10.0 | 0.688897 | 0.000000 | 0.0 | 1.0 | 0.926372 | 0.498361 | 10.0 | 7.0 |
| 2020-02-01 | 0.938151 | 0.016057 | 0.0 | 4.0 | 0.472478 | 0.000000 | 0.0 | 1.0 | 0.739700 | 0.030127 | 1.0 | 1.0 | 0.314258 | 0.044444 | 1.0 | 1.0 | 0.834762 | 0.048253 | 2.0 | 1.0 | 0.855825 | 0.177616 | 1.0 | 2.0 | 0.840946 | 0.015929 | 0.0 | 4.0 | 0.910804 | 0.110666 | 2.0 | 1.0 | 0.858063 | 0.023241 | 3.0 | 1.0 | 0.853967 | 0.010243 | 0.0 | 7.0 | 0.954210 | 0.233401 | 1.0 | 5.0 | 0.852730 | 0.083917 | 4.0 | 1.0 | 0.740020 | 0.217472 | 5.0 | 1.0 | 0.618056 | 0.008397 | 6.0 | 1.0 | 0.955443 | 0.520869 | 2.0 | 8.0 | 0.841128 | 0.010969 | 0.0 | 2.0 | 0.956256 | 0.306333 | 0.0 | 8.0 | 0.993463 | 0.121406 | 1.0 | 4.0 | 0.978786 | 0.197812 | 2.0 | 3.0 | 0.923564 | 0.262506 | 1.0 | 8.0 | 0.754559 | 0.005433 | 0.0 | 3.0 | 0.820884 | 0.453092 | 0.0 | 10.0 | 0.733067 | 0.071864 | 0.0 | 5.0 | 0.953285 | 0.517918 | 3.0 | 4.0 | 0.939391 | 0.545588 | 1.0 | 10.0 | 0.960495 | 0.686631 | 2.0 | 10.0 | 0.949298 | 0.827015 | 5.0 | 5.0 | 0.922440 | 0.252982 | 7.0 | 1.0 | 0.696099 | 0.000000 | 0.0 | 1.0 | 0.912739 | 0.520170 | 10.0 | 1.0 |
| 2020-02-02 | 0.924102 | 0.021547 | 1.0 | 1.0 | 0.580662 | 0.112383 | 7.0 | 1.0 | 0.743684 | 0.032635 | 1.0 | 1.0 | 0.287863 | 0.041030 | 1.0 | 1.0 | 0.839652 | 0.042153 | 2.0 | 1.0 | 0.858061 | 0.175067 | 1.0 | 2.0 | 0.845195 | 0.018746 | 0.0 | 4.0 | 0.894481 | 0.091355 | 1.0 | 2.0 | 0.872405 | 0.043329 | 3.0 | 1.0 | 0.885565 | 0.029885 | 0.0 | 8.0 | 0.956351 | 0.224820 | 1.0 | 5.0 | 0.851522 | 0.080107 | 4.0 | 1.0 | 0.830691 | 0.225732 | 8.0 | 9.0 | 0.663796 | 0.041563 | 6.0 | 1.0 | 0.958060 | 0.477648 | 2.0 | 8.0 | 0.794370 | 0.086173 | 0.0 | 10.0 | 0.956778 | 0.295403 | 1.0 | 6.0 | 0.993012 | 0.126859 | 0.0 | 6.0 | 0.977680 | 0.195274 | 2.0 | 3.0 | 0.929984 | 0.277312 | 1.0 | 10.0 | 0.745377 | 0.072809 | 0.0 | 10.0 | 0.828744 | 0.450976 | 0.0 | 10.0 | 0.742354 | 0.071990 | 0.0 | 5.0 | 0.949350 | 0.511481 | 4.0 | 1.0 | 0.938758 | 0.569939 | 2.0 | 10.0 | 0.964764 | 0.631864 | 2.0 | 10.0 | 0.952497 | 0.827609 | 6.0 | 4.0 | 0.937082 | 0.294044 | 3.0 | 10.0 | 0.695605 | 0.000000 | 0.0 | 1.0 | 0.866263 | 0.457535 | 10.0 | 1.0 |
| 2020-02-03 | 0.897889 | 0.011127 | 0.0 | 2.0 | 0.578586 | 0.084602 | 6.0 | 1.0 | 0.751122 | 0.036481 | 1.0 | 1.0 | 0.264429 | 0.034921 | 1.0 | 1.0 | 0.840418 | 0.040969 | 2.0 | 1.0 | 0.857816 | 0.176034 | 1.0 | 2.0 | 0.849143 | 0.019537 | 0.0 | 4.0 | 0.886386 | 0.107646 | 1.0 | 2.0 | 0.883953 | 0.073585 | 3.0 | 2.0 | 0.842666 | 0.217758 | 1.0 | 2.0 | 0.948789 | 0.245775 | 1.0 | 5.0 | 0.857931 | 0.077984 | 4.0 | 1.0 | 0.908256 | 0.442118 | 10.0 | 3.0 | 0.769221 | 0.211021 | 6.0 | 3.0 | 0.958466 | 0.442785 | 2.0 | 9.0 | 0.775948 | 0.066843 | 0.0 | 10.0 | 0.954846 | 0.330638 | 1.0 | 6.0 | 0.992010 | 0.136615 | 0.0 | 7.0 | 0.977565 | 0.205029 | 2.0 | 3.0 | 0.933762 | 0.290974 | 0.0 | 10.0 | 0.687029 | 0.024173 | 2.0 | 1.0 | 0.834124 | 0.467381 | 0.0 | 10.0 | 0.755553 | 0.093837 | 3.0 | 1.0 | 0.947039 | 0.519875 | 3.0 | 4.0 | 0.944696 | 0.620016 | 3.0 | 10.0 | 0.944922 | 0.604672 | 3.0 | 10.0 | 0.939818 | 0.891324 | 4.0 | 10.0 | 0.940012 | 0.472107 | 6.0 | 10.0 | 0.707043 | 0.076362 | 7.0 | 1.0 | 0.875319 | 0.537691 | 10.0 | 1.0 |
| 2020-02-04 | 0.904921 | 0.031307 | 1.0 | 1.0 | 0.597198 | 0.104456 | 6.0 | 1.0 | 0.744069 | 0.029407 | 1.0 | 1.0 | 0.246631 | 0.020256 | 1.0 | 1.0 | 0.823586 | 0.026186 | 0.0 | 3.0 | 0.849725 | 0.168015 | 1.0 | 2.0 | 0.825145 | 0.000000 | 0.0 | 1.0 | 0.887502 | 0.125386 | 1.0 | 2.0 | 0.887025 | 0.078661 | 3.0 | 2.0 | 0.851339 | 0.251436 | 2.0 | 1.0 | 0.948959 | 0.258524 | 1.0 | 5.0 | 0.854753 | 0.114329 | 4.0 | 1.0 | 0.939236 | 0.584200 | 10.0 | 2.0 | 0.765893 | 0.189358 | 6.0 | 1.0 | 0.961577 | 0.411895 | 2.0 | 9.0 | 0.747376 | 0.037753 | 0.0 | 7.0 | 0.953472 | 0.342033 | 1.0 | 6.0 | 0.992247 | 0.163202 | 0.0 | 7.0 | 0.970482 | 0.228584 | 2.0 | 3.0 | 0.934823 | 0.313894 | 0.0 | 10.0 | 0.695605 | 0.116481 | 0.0 | 10.0 | 0.836810 | 0.499108 | 0.0 | 10.0 | 0.751535 | 0.158736 | 3.0 | 1.0 | 0.946863 | 0.526888 | 3.0 | 4.0 | 0.942425 | 0.633289 | 2.0 | 10.0 | 0.949180 | 0.574617 | 3.0 | 10.0 | 0.946641 | 0.928926 | 4.0 | 10.0 | 0.956384 | 0.614698 | 6.0 | 10.0 | 0.715470 | 0.089890 | 0.0 | 8.0 | 0.872729 | 0.595870 | 5.0 | 1.0 |
| 2020-02-05 | 0.906931 | 0.032411 | 0.0 | 3.0 | 0.559343 | 0.067878 | 6.0 | 1.0 | 0.729910 | 0.033307 | 1.0 | 1.0 | 0.242735 | 0.016471 | 1.0 | 1.0 | 0.823482 | 0.028457 | 0.0 | 3.0 | 0.849644 | 0.175001 | 1.0 | 2.0 | 0.744241 | 0.064680 | 2.0 | 1.0 | 0.886492 | 0.141837 | 1.0 | 2.0 | 0.882650 | 0.098079 | 3.0 | 2.0 | 0.827581 | 0.238905 | 1.0 | 4.0 | 0.950287 | 0.269456 | 1.0 | 5.0 | 0.839329 | 0.106574 | 4.0 | 1.0 | 0.862363 | 0.516535 | 6.0 | 6.0 | 0.811936 | 0.148159 | 6.0 | 1.0 | 0.955354 | 0.399661 | 2.0 | 8.0 | 0.751067 | 0.065501 | 0.0 | 7.0 | 0.953734 | 0.357351 | 1.0 | 6.0 | 0.991581 | 0.186797 | 0.0 | 7.0 | 0.893027 | 0.269188 | 1.0 | 6.0 | 0.935135 | 0.355875 | 1.0 | 10.0 | 0.706560 | 0.143205 | 0.0 | 10.0 | 0.825124 | 0.582168 | 1.0 | 9.0 | 0.754968 | 0.247058 | 3.0 | 1.0 | 0.881892 | 0.558555 | 3.0 | 4.0 | 0.945325 | 0.652020 | 3.0 | 10.0 | 0.952449 | 0.538502 | 4.0 | 10.0 | 0.943961 | 0.946659 | 4.0 | 10.0 | 0.966246 | 0.729846 | 6.0 | 10.0 | 0.741169 | 0.160325 | 0.0 | 10.0 | 0.864186 | 0.606410 | 5.0 | 1.0 |
| 2020-02-06 | 0.903362 | 0.031909 | 0.0 | 3.0 | 0.496007 | 0.001211 | 0.0 | 7.0 | 0.729779 | 0.034584 | 1.0 | 1.0 | 0.241851 | 0.012645 | 1.0 | 1.0 | 0.823624 | 0.030115 | 0.0 | 3.0 | 0.842313 | 0.180065 | 1.0 | 2.0 | 0.734758 | 0.075714 | 2.0 | 2.0 | 0.883214 | 0.149839 | 1.0 | 3.0 | 0.876524 | 0.122025 | 3.0 | 2.0 | 0.835168 | 0.269057 | 1.0 | 4.0 | 0.950327 | 0.267850 | 1.0 | 5.0 | 0.820854 | 0.087590 | 4.0 | 1.0 | 0.858126 | 0.525595 | 7.0 | 2.0 | 0.690856 | 0.023524 | 6.0 | 1.0 | 0.956503 | 0.378440 | 2.0 | 8.0 | 0.752592 | 0.092564 | 0.0 | 8.0 | 0.951657 | 0.377785 | 1.0 | 6.0 | 0.987199 | 0.216250 | 0.0 | 8.0 | 0.878649 | 0.308021 | 0.0 | 10.0 | 0.933603 | 0.373989 | 0.0 | 10.0 | 0.715242 | 0.173652 | 0.0 | 10.0 | 0.792536 | 0.527570 | 0.0 | 10.0 | 0.749571 | 0.310903 | 3.0 | 1.0 | 0.879474 | 0.585705 | 3.0 | 4.0 | 0.940682 | 0.691872 | 3.0 | 10.0 | 0.949048 | 0.522331 | 3.0 | 10.0 | 0.946996 | 0.970302 | 4.0 | 10.0 | 0.970741 | 0.876423 | 7.0 | 10.0 | 0.747141 | 0.186050 | 0.0 | 10.0 | 0.858789 | 0.651210 | 5.0 | 1.0 |
| 2020-02-07 | 0.888820 | 0.030485 | 0.0 | 3.0 | 0.497183 | 0.000000 | 0.0 | 1.0 | 0.729205 | 0.038121 | 1.0 | 1.0 | 0.244023 | 0.013567 | 1.0 | 1.0 | 0.806110 | 0.035931 | 0.0 | 3.0 | 0.844450 | 0.176980 | 1.0 | 2.0 | 0.733922 | 0.079774 | 2.0 | 2.0 | 0.881972 | 0.153232 | 1.0 | 3.0 | 0.834812 | 0.119702 | 3.0 | 2.0 | 0.829037 | 0.269289 | 0.0 | 5.0 | 0.948801 | 0.265431 | 1.0 | 5.0 | 0.814548 | 0.081401 | 0.0 | 6.0 | 0.834411 | 0.509857 | 6.0 | 3.0 | 0.669247 | 0.000000 | 0.0 | 1.0 | 0.916988 | 0.368660 | 6.0 | 3.0 | 0.753504 | 0.113608 | 0.0 | 8.0 | 0.951581 | 0.387047 | 1.0 | 6.0 | 0.985964 | 0.231457 | 0.0 | 8.0 | 0.879896 | 0.331863 | 0.0 | 10.0 | 0.932057 | 0.413848 | 1.0 | 9.0 | 0.702435 | 0.159992 | 0.0 | 10.0 | 0.793570 | 0.555758 | 1.0 | 9.0 | 0.737929 | 0.378830 | 3.0 | 10.0 | 0.820114 | 0.611131 | 3.0 | 5.0 | 0.939164 | 0.724347 | 3.0 | 10.0 | 0.948532 | 0.509251 | 3.0 | 10.0 | 0.937392 | 0.964968 | 4.0 | 10.0 | 0.919110 | 0.839670 | 6.0 | 8.0 | 0.753460 | 0.223537 | 0.0 | 10.0 | 0.848225 | 0.732779 | 4.0 | 8.0 |
| 2020-02-08 | 0.869338 | 0.037199 | 1.0 | 1.0 | 0.499039 | 0.000000 | 0.0 | 1.0 | 0.714344 | 0.047336 | 1.0 | 1.0 | 0.244616 | 0.012636 | 1.0 | 1.0 | 0.802236 | 0.036244 | 0.0 | 3.0 | 0.847168 | 0.173278 | 1.0 | 2.0 | 0.728124 | 0.062409 | 2.0 | 2.0 | 0.883384 | 0.151027 | 1.0 | 3.0 | 0.795838 | 0.089461 | 3.0 | 2.0 | 0.826822 | 0.265795 | 1.0 | 3.0 | 0.942943 | 0.271136 | 1.0 | 5.0 | 0.817268 | 0.097057 | 4.0 | 1.0 | 0.848523 | 0.594500 | 6.0 | 3.0 | 0.660238 | 0.022379 | 6.0 | 1.0 | 0.895003 | 0.357835 | 7.0 | 1.0 | 0.734552 | 0.194497 | 6.0 | 1.0 | 0.946219 | 0.405826 | 1.0 | 6.0 | 0.985018 | 0.238531 | 0.0 | 8.0 | 0.879558 | 0.358587 | 0.0 | 10.0 | 0.910049 | 0.483997 | 1.0 | 10.0 | 0.694411 | 0.152365 | 0.0 | 10.0 | 0.793455 | 0.591248 | 1.0 | 9.0 | 0.732615 | 0.386708 | 2.0 | 10.0 | 0.820699 | 0.622035 | 3.0 | 5.0 | 0.938653 | 0.736543 | 3.0 | 10.0 | 0.936375 | 0.495513 | 3.0 | 10.0 | 0.936184 | 0.971521 | 4.0 | 9.0 | 0.870373 | 0.788380 | 4.0 | 10.0 | 0.758244 | 0.260710 | 0.0 | 10.0 | 0.850728 | 0.765206 | 5.0 | 4.0 |
| 2020-02-09 | 0.785775 | 0.029803 | 0.0 | 3.0 | 0.507021 | 0.000000 | 0.0 | 1.0 | 0.716084 | 0.042191 | 1.0 | 1.0 | 0.245538 | 0.014661 | 1.0 | 1.0 | 0.798145 | 0.038738 | 0.0 | 3.0 | 0.849358 | 0.168122 | 1.0 | 2.0 | 0.690819 | 0.092063 | 2.0 | 2.0 | 0.885131 | 0.146606 | 1.0 | 3.0 | 0.769118 | 0.070005 | 3.0 | 2.0 | 0.826082 | 0.266649 | 1.0 | 3.0 | 0.924345 | 0.275969 | 1.0 | 5.0 | 0.800861 | 0.084155 | 0.0 | 6.0 | 0.818504 | 0.555519 | 6.0 | 1.0 | 0.644573 | 0.005736 | 6.0 | 1.0 | 0.866306 | 0.331306 | 6.0 | 3.0 | 0.733554 | 0.212494 | 6.0 | 1.0 | 0.945656 | 0.416247 | 1.0 | 7.0 | 0.985013 | 0.245401 | 0.0 | 8.0 | 0.879850 | 0.369754 | 0.0 | 10.0 | 0.899286 | 0.470447 | 1.0 | 10.0 | 0.695125 | 0.179075 | 0.0 | 10.0 | 0.780473 | 0.643443 | 1.0 | 10.0 | 0.736647 | 0.426230 | 2.0 | 10.0 | 0.820448 | 0.638850 | 3.0 | 5.0 | 0.939663 | 0.755586 | 3.0 | 10.0 | 0.909569 | 0.475412 | 3.0 | 10.0 | 0.929921 | 0.963563 | 4.0 | 9.0 | 0.872288 | 0.825719 | 4.0 | 10.0 | 0.750313 | 0.317793 | 0.0 | 10.0 | 0.839755 | 0.839877 | 4.0 | 10.0 |
| 2020-02-10 | 0.753200 | 0.007857 | 0.0 | 3.0 | 0.509212 | 0.000000 | 0.0 | 1.0 | 0.719142 | 0.042368 | 1.0 | 1.0 | 0.243930 | 0.011547 | 1.0 | 1.0 | 0.800754 | 0.037771 | 0.0 | 3.0 | 0.834206 | 0.169422 | 1.0 | 2.0 | 0.649933 | 0.100935 | 2.0 | 2.0 | 0.886431 | 0.144583 | 1.0 | 3.0 | 0.733127 | 0.040063 | 3.0 | 2.0 | 0.810431 | 0.260889 | 1.0 | 3.0 | 0.895751 | 0.255645 | 2.0 | 4.0 | 0.793658 | 0.074994 | 0.0 | 6.0 | 0.805994 | 0.527903 | 6.0 | 1.0 | 0.637053 | 0.000000 | 0.0 | 1.0 | 0.829212 | 0.301897 | 7.0 | 1.0 | 0.730961 | 0.213704 | 6.0 | 1.0 | 0.944656 | 0.410136 | 1.0 | 7.0 | 0.984924 | 0.260041 | 0.0 | 8.0 | 0.877359 | 0.389465 | 1.0 | 10.0 | 0.888775 | 0.452585 | 1.0 | 9.0 | 0.668043 | 0.146244 | 0.0 | 10.0 | 0.775507 | 0.613323 | 1.0 | 9.0 | 0.734787 | 0.434014 | 2.0 | 10.0 | 0.782331 | 0.668174 | 3.0 | 6.0 | 0.929784 | 0.742485 | 3.0 | 10.0 | 0.870947 | 0.442062 | 3.0 | 10.0 | 0.926472 | 0.960706 | 4.0 | 9.0 | 0.876944 | 0.878580 | 4.0 | 10.0 | 0.729089 | 0.433173 | 3.0 | 10.0 | 0.844804 | 0.882167 | 4.0 | 10.0 |
| 2020-02-11 | 0.747769 | 0.000000 | 0.0 | 1.0 | 0.516061 | 0.000000 | 0.0 | 1.0 | 0.720783 | 0.043738 | 1.0 | 1.0 | 0.244932 | 0.009625 | 1.0 | 1.0 | 0.804601 | 0.037236 | 0.0 | 3.0 | 0.832009 | 0.171897 | 1.0 | 2.0 | 0.576981 | 0.107064 | 2.0 | 2.0 | 0.888260 | 0.140159 | 1.0 | 3.0 | 0.715380 | 0.022356 | 3.0 | 2.0 | 0.807888 | 0.254900 | 0.0 | 5.0 | 0.883705 | 0.244150 | 0.0 | 6.0 | 0.790292 | 0.069632 | 0.0 | 6.0 | 0.801333 | 0.516260 | 6.0 | 1.0 | 0.631481 | 0.092614 | 6.0 | 2.0 | 0.797204 | 0.271135 | 7.0 | 1.0 | 0.712333 | 0.171781 | 0.0 | 8.0 | 0.940236 | 0.422466 | 1.0 | 7.0 | 0.978752 | 0.294292 | 0.0 | 9.0 | 0.868370 | 0.369639 | 0.0 | 10.0 | 0.876497 | 0.422796 | 0.0 | 10.0 | 0.666962 | 0.145386 | 0.0 | 10.0 | 0.776235 | 0.606210 | 1.0 | 9.0 | 0.722189 | 0.400684 | 1.0 | 10.0 | 0.781742 | 0.683850 | 2.0 | 10.0 | 0.921341 | 0.729756 | 3.0 | 10.0 | 0.832147 | 0.405049 | 3.0 | 10.0 | 0.926659 | 0.971797 | 4.0 | 9.0 | 0.874191 | 0.888166 | 4.0 | 10.0 | 0.737170 | 0.488307 | 4.0 | 10.0 | 0.835791 | 0.938297 | 5.0 | 10.0 |

In [10]:

```
# table 2
df = df_move_inc_corr.loc[['2020-01-21', '2020-01-24', '2020-01-27', '2020-01-30', '2020-02-02', 
                           '2020-02-05', '2020-02-08', '2020-02-11'], mentioned_regions]
cols = np.array([2, 3])
idx = []
for i in range(len(mentioned_regions)):
    idx += (cols + i * 4).tolist()
df = df.iloc[:, idx].astype(object)
for i, row in enumerate(df.values):
    for j, val in enumerate(row):
        if ~np.isnan(val):
            df.iloc[i, j] = int(val)
df.columns = pd.MultiIndex.from_product([['Shanghai', 'Liaoning', 'Zhejiang', 'Beijing', 'Jilin', 'Tianjin', 
                                          'Sichuan', 'Jiangsu', 'Anhui', 'Henan', 'Heilongjiang'], 
                                         ['OFF SET', 'WIN DOW']])
df.index = ['{}/{}/{}'.format(date[5:7], date[-2:], date[:4]) for date in df.index]
df.to_excel('table2.xlsx')
df2 = df
df2
```

Out[10]:

|  | Shanghai | | Liaoning | | Zhejiang | | Beijing | | Jilin | | Tianjin | | Sichuan | | Jiangsu | | Anhui | | Henan | | Heilongjiang | |
| --- | --- | --- | --- | --- | --- | --- | --- | --- | --- | --- | --- | --- | --- | --- | --- | --- | --- | --- | --- | --- | --- | --- |
|  | OFF SET | WIN DOW | OFF SET | WIN DOW | OFF SET | WIN DOW | OFF SET | WIN DOW | OFF SET | WIN DOW | OFF SET | WIN DOW | OFF SET | WIN DOW | OFF SET | WIN DOW | OFF SET | WIN DOW | OFF SET | WIN DOW | OFF SET | WIN DOW |
| 01/21/2020 | 3 | 1 | NaN | NaN | 4 | 1 | 1 | 2 | NaN | NaN | 4 | 1 | NaN | NaN | NaN | NaN | NaN | NaN | 4 | 1 | NaN | NaN |
| 01/24/2020 | 3 | 1 | 7 | 3 | 2 | 1 | 2 | 1 | 2 | 1 | 1 | 1 | 1 | 4 | 2 | 1 | 3 | 2 | 6 | 7 | 2 | 5 |
| 01/27/2020 | 6 | 1 | 1 | 1 | 2 | 1 | 4 | 2 | 0 | 1 | 1 | 1 | 2 | 1 | 1 | 4 | 0 | 10 | 4 | 1 | 0 | 10 |
| 01/30/2020 | 6 | 1 | 1 | 1 | 2 | 1 | 0 | 1 | 2 | 1 | 0 | 1 | 0 | 10 | 1 | 4 | 4 | 2 | 2 | 10 | 0 | 10 |
| 02/02/2020 | 1 | 1 | 1 | 1 | 1 | 2 | 0 | 8 | 8 | 9 | 6 | 1 | 1 | 6 | 0 | 6 | 2 | 10 | 2 | 10 | 3 | 10 |
| 02/05/2020 | 0 | 3 | 1 | 1 | 1 | 2 | 1 | 4 | 6 | 6 | 6 | 1 | 1 | 6 | 0 | 7 | 3 | 10 | 4 | 10 | 6 | 10 |
| 02/08/2020 | 1 | 1 | 1 | 1 | 1 | 3 | 1 | 3 | 6 | 3 | 6 | 1 | 1 | 6 | 0 | 8 | 3 | 10 | 3 | 10 | 4 | 10 |
| 02/11/2020 | 0 | 1 | 1 | 1 | 1 | 3 | 0 | 5 | 6 | 1 | 6 | 2 | 1 | 7 | 0 | 9 | 3 | 10 | 3 | 10 | 4 | 10 |

In [11]:

```
analyzer.df_virus_daily_injured.loc[['2020-02-11'], ['Shanghai', 'Beijing', 'Tianjin']]
```

Out[11]:

|  | Shanghai | Beijing | Tianjin |
| --- | --- | --- | --- |
| date |  |  |  |
| 2020-02-11 | 306 | 352 | 106 |

In [12]:

```
# table 3
columns = pd.MultiIndex.from_product([
    ['Shanghai cumulative confirmed cases', 'Beijing cumulative confirmed cases', 'Tianjin cumulative confirmed cases'], 
    ['incomming immigrants', 'local residents', 'local infection rate(%)']])
df = pd.DataFrame([
    [99, 306-99, int((306-99)/306*1000) / 10.0, 25, 352, int(352/(25+352)*1000) / 10.0, 6, 106, int(106/(6+106)*1000) / 10.0],
#     [111, 225, 225/(111+225)*100, 25, 375, 375/(25+375)*100, 6, 129, 129/(6+129)*100]
], columns=columns, index=['until 02/11/2020'])  # , 'until 2020-02-25'
df.to_excel('table3.xlsx')
df3 = df
df3
```

Out[12]:

|  | Shanghai cumulative confirmed cases | | | Beijing cumulative confirmed cases | | | Tianjin cumulative confirmed cases | | |
| --- | --- | --- | --- | --- | --- | --- | --- | --- | --- |
|  | incomming immigrants | local residents | local infection rate(%) | incomming immigrants | local residents | local infection rate(%) | incomming immigrants | local residents | local infection rate(%) |
| until 02/11/2020 | 99 | 207 | 67.6 | 25 | 352 | 93.3 | 6 | 106 | 94.6 |

In [13]:

```
# table 4
df_move_inc_corr = analyzer.df_move_inc_corr.loc[['2020-02-10']]
# R0 data is from reference
r0 = {'Shanghai': 0.46, 'Zhejiang': 0.52, 'Jiangsu': 0.82, 'Anhui': 0.98, 'Henan': 0.75, 'Sichuan': 0.81}
regions = list(r0)
# df = df_move_inc_corr[list(r0)]
# df_r0 = pd.DataFrame([r0], index=df.index)
# df_r0.columns = pd.MultiIndex.from_product([df_r0.columns, ['R0']])
# df = pd.concat([df_r0, df_move_inc_corr[list(r0)]], axis=1, sort=False).sort_index(axis=1)[regions]
# df_mean = pd.DataFrame(df.iloc[:, 2::4].values + df.iloc[:, 3::4].values, 
#                        index=df.index, columns=pd.MultiIndex.from_product([regions, ['offset + window']]))
# df = pd.concat([df.iloc[:, 0::4], df_mean, ], axis=1).sort_index(axis=1)  # df.iloc[:, 2::4], df.iloc[:, 3::4]
# df[['Shanghai', 'Zhejiang', 'Sichuan', 'Jiangsu', 'Henan', 'Anhui']]
df = pd.DataFrame([r0])
df = df[['Shanghai', 'Zhejiang', 'Sichuan', 'Jiangsu', 'Henan', 'Anhui']]
df.to_excel('table4.xlsx')
df4 = df
df4
```

Out[13]:

|  | Shanghai | Zhejiang | Sichuan | Jiangsu | Henan | Anhui |
| --- | --- | --- | --- | --- | --- | --- |
| 0 | 0.46 | 0.52 | 0.81 | 0.82 | 0.75 | 0.98 |

In [14]:

```
regions = ['Shanghai', 'Beijing', 'Tianjin']
df = df2[regions]
df = pd.DataFrame([df[region].sum(axis=1) for region in regions], 
                  index=['{} OFFSET + WINDOW'.format(r) for r in regions]).T.iloc[-2:]
df3_offset_window = df.astype(np.int32)
df3_offset_window
```

Out[14]:

|  | Shanghai OFFSET + WINDOW | Beijing OFFSET + WINDOW | Tianjin OFFSET + WINDOW |
| --- | --- | --- | --- |
| 02/08/2020 | 2 | 4 | 7 |
| 02/11/2020 | 1 | 5 | 8 |

In [15]:

```
df3_rate = df3.iloc[:, 2::3]
df3_rate
```

Out[15]:

|  | Shanghai cumulative confirmed cases | Beijing cumulative confirmed cases | Tianjin cumulative confirmed cases |
| --- | --- | --- | --- |
|  | local infection rate(%) | local infection rate(%) | local infection rate(%) |
| until 02/11/2020 | 67.6 | 93.3 | 94.6 |

In [16]:

```
corrs = []
for i in range(df3_offset_window.shape[0]):
    corrs.append(np.corrcoef(df3_offset_window.values[i, :], df3_rate.values)[1, 0])
pd.DataFrame([corrs], index=['Correlation coefficient between OFFSET + WINDOW and local infection rate'], 
             columns=df3_offset_window.index).T
```

Out[16]:

|  | Correlation coefficient between OFFSET + WINDOW and local infection rate |
| --- | --- |
| 02/08/2020 | 0.827666 |
| 02/11/2020 | 0.921603 |

In [17]:

```
regions = df4.columns
df = df2[regions]
df = pd.DataFrame([df[region].sum(axis=1) for region in regions], 
                  index=['{} OFFSET + WINDOW'.format(r) for r in regions]).T.iloc[-2:]
df4_offset_window = df.astype(np.int32)
df4_offset_window
```

Out[17]:

|  | Shanghai OFFSET + WINDOW | Zhejiang OFFSET + WINDOW | Sichuan OFFSET + WINDOW | Jiangsu OFFSET + WINDOW | Henan OFFSET + WINDOW | Anhui OFFSET + WINDOW |
| --- | --- | --- | --- | --- | --- | --- |
| 02/08/2020 | 2 | 4 | 7 | 8 | 13 | 13 |
| 02/11/2020 | 1 | 4 | 8 | 9 | 13 | 13 |

In [18]:

```
corrs = []
for i in range(df4_offset_window.shape[0]):
    corrs.append(np.corrcoef(df4_offset_window.values[i, :], df4.values)[1, 0])
pd.DataFrame([corrs], index=['Correlation coefficient between OFFSET + WINDOW and R0'], 
             columns=df4_offset_window.index).T
```

Out[18]:

|  | Correlation coefficient between OFFSET + WINDOW and R0 |
| --- | --- |
| 02/08/2020 | 0.833326 |
| 02/11/2020 | 0.878733 |

In [19]:

```
index = pd.Index(['Heilongjiang', 'Jilin', 'Liaoning'], name='REGION')
df5 = pd.DataFrame({'confident': [4.1, 3.9, 3.7],
                   'alert': [3.8, 3.9, 3.9],
                   'scared': [2.1, 2.2, 2.3],
                  }, index=index)
df5['alert + scared - confident'] = df5['alert'] + df5['scared'] - df5['confident']
df5.to_excel('table5.xlsx')
df5
```

Out[19]:

|  | confident | alert | scared | alert + scared - confident |
| --- | --- | --- | --- | --- |
| REGION |  |  |  |  |
| Heilongjiang | 4.1 | 3.8 | 2.1 | 1.8 |
| Jilin | 3.9 | 3.9 | 2.2 | 2.2 |
| Liaoning | 3.7 | 3.9 | 2.3 | 2.5 |

In [20]:

```
regions = df5.index
df = df2[regions]
df = pd.DataFrame([df[region].sum(axis=1) for region in regions], 
                  index=['{} OFFSET + WINDOW'.format(r) for r in regions]).T.iloc[2:]
df5_offset_window = df.astype(np.int32)
df5_offset_window
```

Out[20]:

|  | Heilongjiang OFFSET + WINDOW | Jilin OFFSET + WINDOW | Liaoning OFFSET + WINDOW |
| --- | --- | --- | --- |
| 01/27/2020 | 10 | 1 | 2 |
| 01/30/2020 | 10 | 3 | 2 |
| 02/02/2020 | 13 | 17 | 2 |
| 02/05/2020 | 16 | 12 | 2 |
| 02/08/2020 | 14 | 9 | 2 |
| 02/11/2020 | 14 | 7 | 2 |

In [21]:

```
from collections import OrderedDict
dict_corrs = OrderedDict()
for j, col in enumerate(df5.columns):
    dict_corrs[col] = []
    for i in range(df5_offset_window.shape[0]):
        corr = np.corrcoef(df5_offset_window.values[i, :], df5.values[:, j])[1, 0]
        dict_corrs[col].append(corr)
df = pd.DataFrame(dict_corrs, index=df5_offset_window.index)
df.columns = ['Correlation coefficient between OFFSET + WINDOW and {}'.format(col) for col in df.columns]
df
```

Out[21]:

|  | Correlation coefficient between OFFSET + WINDOW and confident | Correlation coefficient between OFFSET + WINDOW and alert | Correlation coefficient between OFFSET + WINDOW and scared | Correlation coefficient between OFFSET + WINDOW and alert + scared - confident |
| --- | --- | --- | --- | --- |
| 01/27/2020 | 0.810885 | -0.994850 | -0.810885 | -0.856244 |
| 01/30/2020 | 0.917663 | -0.993399 | -0.917663 | -0.947220 |
| 02/02/2020 | 0.708083 | -0.260153 | -0.708083 | -0.647643 |
| 02/05/2020 | 0.970725 | -0.720577 | -0.970725 | -0.947697 |
| 02/08/2020 | 0.995402 | -0.814152 | -0.995402 | -0.984160 |
| 02/11/2020 | 0.995402 | -0.909935 | -0.995402 | -0.999907 |

In [22]:

```
def t(n, corr):
    return corr * (((n - 2) / (1 - corr ** 2)) ** 0.5)
```

In [23]:

```
# calculate t by the data in table 2 and table 3
t(3, 0.921603)  # t0.15,1 = 1.963   t0.1,1 = 3.078
```

Out[23]:

```
2.3744461450910754
```

In [24]:

```
# calculate t by the data in table 2 and table 4
t(6, 0.833326), t(6, 0.878733)  # t0.025,4 = 2.776   t0.01,4 = 3.747
```

Out[24]:

```
(3.0150266131153614, 3.681994056186803)
```

In [25]:

```
# calculate t by the data in table 2 and table 5
# t0.15,1 = 1.963   t0.1,1 = 3.078    t0.05,1 = 6.314   t0.005,1 = 63.66
t(3, -0.947697), t(3, -0.984160), t(3, -0.999907)
```

Out[25]:

```
(-2.9692414065895707, -5.551364910762611, -73.31844309343555)
```

In [ ]:

```

```

## Plot "new" and "risk" in all 30 regions under offset=0, window=1¶

## And plot "new" and "processed risk" under best fit by the maximize the "corr"¶

total 60 figures, regions are sorted by effectiveness from best to worst.

In [26]:

```
last_date = '2020-02-11'
analyzer = CoronavirusAnalyzer(last_date=last_date, first_date='2020-01-17', in_english=True)
```

In [27]:

```
analyzer.plot_move_inc_corr('Qinghai', use_best_fit=False)
```

```
offset = 0, window = 1, corr = 0.5160606078898728
```

In [28]:

```
analyzer.plot_move_inc_corr('Qinghai', use_best_fit=True)
```

```
offset = 0, window = 1, corr = 0.5160606078898728
```

In [29]:

```
analyzer.plot_move_inc_corr('Shanghai', use_best_fit=False)
```

```
offset = 0, window = 1, corr = 0.7477691282696345
```

In [30]:

```
analyzer.plot_move_inc_corr('Shanghai', use_best_fit=True)
```

```
offset = 0, window = 1, corr = 0.7477691282696345
```

In [31]:

```
analyzer.plot_move_inc_corr('Liaoning', use_best_fit=False)
```

```
offset = 0, window = 1, corr = 0.6770451612410725
```

In [32]:

```
analyzer.plot_move_inc_corr('Liaoning', use_best_fit=True)
```

```
offset = 1, window = 1, corr = 0.7207829657404504
```

In [33]:

```
analyzer.plot_move_inc_corr('Xizang', use_best_fit=False)
```

```
offset = 0, window = 1, corr = 0.2353077695414271
```

In [34]:

```
analyzer.plot_move_inc_corr('Xizang', use_best_fit=True)
```

```
offset = 1, window = 1, corr = 0.24493238818539234
```

In [35]:

```
analyzer.plot_move_inc_corr('Fujian', use_best_fit=False)
```

```
offset = 0, window = 1, corr = 0.7673646826509236
```

In [36]:

```
analyzer.plot_move_inc_corr('Fujian', use_best_fit=True)
```

```
offset = 0, window = 3, corr = 0.8046008415533149
```

In [37]:

```
analyzer.plot_move_inc_corr('Yunnan', use_best_fit=False)
```

```
offset = 0, window = 1, corr = 0.6601123386102973
```

In [38]:

```
analyzer.plot_move_inc_corr('Yunnan', use_best_fit=True)
```

```
offset = 1, window = 2, corr = 0.8320089333919763
```

In [39]:

```
analyzer.plot_move_inc_corr('Zhejiang', use_best_fit=False)
```

```
offset = 0, window = 1, corr = 0.7481007520525306
```

In [40]:

```
analyzer.plot_move_inc_corr('Zhejiang', use_best_fit=True)
```

```
offset = 1, window = 3, corr = 0.8882597508817133
```

In [41]:

```
analyzer.plot_move_inc_corr('Beijing', use_best_fit=False)
```

```
offset = 0, window = 1, corr = 0.5529875974557777
```

In [42]:

```
analyzer.plot_move_inc_corr('Beijing', use_best_fit=True)
```

```
offset = 0, window = 5, corr = 0.8078879832688707
```

In [43]:

```
analyzer.plot_move_inc_corr('Chongqing', use_best_fit=False)
```

```
offset = 0, window = 1, corr = 0.693023695871494
```

In [44]:

```
analyzer.plot_move_inc_corr('Chongqing', use_best_fit=True)
```

```
offset = 3, window = 2, corr = 0.7153797460021183
```

In [45]:

```
analyzer.plot_move_inc_corr('Shaanxi', use_best_fit=False)
```

```
offset = 0, window = 1, corr = 0.7206600345174448
```

In [46]:

```
analyzer.plot_move_inc_corr('Shaanxi', use_best_fit=True)
```

```
offset = 0, window = 6, corr = 0.7902919861777367
```

In [47]:

```
analyzer.plot_move_inc_corr('Guangdong', use_best_fit=False)
```

```
offset = 0, window = 1, corr = 0.6395545857715658
```

In [48]:

```
analyzer.plot_move_inc_corr('Guangdong', use_best_fit=True)
```

```
offset = 0, window = 6, corr = 0.8837047463588393
```

In [49]:

```
analyzer.plot_move_inc_corr('Jilin', use_best_fit=False)
```

```
offset = 0, window = 1, corr = 0.2850731393712041
```

In [50]:

```
analyzer.plot_move_inc_corr('Jilin', use_best_fit=True)
```

```
offset = 6, window = 1, corr = 0.8013334240920934
```

In [51]:

```
analyzer.plot_move_inc_corr('Shanxi', use_best_fit=False)
```

```
offset = 0, window = 1, corr = 0.45370108030042283
```

In [52]:

```
analyzer.plot_move_inc_corr('Shanxi', use_best_fit=True)
```

```
offset = 0, window = 10, corr = 0.8764966070906736
```

In [53]:

```
analyzer.plot_move_inc_corr('Sichuan', use_best_fit=False)
```

```
offset = 0, window = 1, corr = 0.5177693495351221
```

In [54]:

```
analyzer.plot_move_inc_corr('Sichuan', use_best_fit=True)
```

```
offset = 1, window = 7, corr = 0.9402355751439356
```

In [55]:

```
analyzer.plot_move_inc_corr('Tianjin', use_best_fit=False)
```

```
offset = 0, window = 1, corr = 0.5388671212412173
```

In [56]:

```
analyzer.plot_move_inc_corr('Tianjin', use_best_fit=True)
```

```
offset = 6, window = 2, corr = 0.6314812676604681
```

In [57]:

```
analyzer.plot_move_inc_corr('Gansu', use_best_fit=False)
```

```
offset = 0, window = 1, corr = 0.5405523215503308
```

In [58]:

```
analyzer.plot_move_inc_corr('Gansu', use_best_fit=True)
```

```
offset = 0, window = 8, corr = 0.7123331825366783
```

In [59]:

```
analyzer.plot_move_inc_corr('Hunan', use_best_fit=False)
```

```
offset = 0, window = 1, corr = 0.5260691650587502
```

In [60]:

```
analyzer.plot_move_inc_corr('Hunan', use_best_fit=True)
```

```
offset = 7, window = 1, corr = 0.7972037271072374
```

In [61]:

```
analyzer.plot_move_inc_corr('Jiangsu', use_best_fit=False)
```

```
offset = 0, window = 1, corr = 0.6844593863310701
```

In [62]:

```
analyzer.plot_move_inc_corr('Jiangsu', use_best_fit=True)
```

```
offset = 0, window = 9, corr = 0.9787517969771452
```

In [63]:

```
analyzer.plot_move_inc_corr('Guangxi', use_best_fit=False)
```

```
offset = 0, window = 1, corr = 0.17002558252159977
```

In [64]:

```
analyzer.plot_move_inc_corr('Guangxi', use_best_fit=True)
```

```
offset = 1, window = 9, corr = 0.7762350832933463
```

In [65]:

```
analyzer.plot_move_inc_corr('Neimenggu', use_best_fit=False)
```

```
offset = 0, window = 1, corr = 0.5215755371313396
```

In [66]:

```
analyzer.plot_move_inc_corr('Neimenggu', use_best_fit=True)
```

```
offset = 0, window = 10, corr = 0.6669619051316951
```

In [67]:

```
analyzer.plot_move_inc_corr('Ningxia', use_best_fit=False)
```

```
offset = 0, window = 1, corr = 0.4699166674750525
```

In [68]:

```
analyzer.plot_move_inc_corr('Ningxia', use_best_fit=True)
```

```
offset = 2, window = 2, corr = 0.5769807688191106
```

In [69]:

```
analyzer.plot_move_inc_corr('Shandong', use_best_fit=False)
```

```
offset = 0, window = 1, corr = 0.49873127781405435
```

In [70]:

```
analyzer.plot_move_inc_corr('Shandong', use_best_fit=True)
```

```
offset = 0, window = 10, corr = 0.8683701390787155
```

In [71]:

```
analyzer.plot_move_inc_corr('Hainan', use_best_fit=False)
```

```
offset = 0, window = 1, corr = 0.321505539480881
```

In [72]:

```
analyzer.plot_move_inc_corr('Hainan', use_best_fit=True)
```

```
offset = 1, window = 10, corr = 0.722189099013955
```

In [73]:

```
analyzer.plot_move_inc_corr('Anhui', use_best_fit=False)
```

```
offset = 0, window = 1, corr = 0.19158500503824055
```

In [74]:

```
analyzer.plot_move_inc_corr('Anhui', use_best_fit=True)
```

```
offset = 3, window = 10, corr = 0.9213411181027489
```

In [75]:

```
analyzer.plot_move_inc_corr('Jiangxi', use_best_fit=False)
```

```
offset = 0, window = 1, corr = -0.0451381321405444
```

In [76]:

```
analyzer.plot_move_inc_corr('Jiangxi', use_best_fit=True)
```

```
offset = 4, window = 9, corr = 0.9266590397317811
```

In [77]:

```
analyzer.plot_move_inc_corr('Henan', use_best_fit=False)
```

```
offset = 0, window = 1, corr = 0.427097620580034
```

In [78]:

```
analyzer.plot_move_inc_corr('Henan', use_best_fit=True)
```

```
offset = 3, window = 10, corr = 0.832146526828929
```

In [79]:

```
analyzer.plot_move_inc_corr('Heilongjiang', use_best_fit=False)
```

```
offset = 0, window = 1, corr = -0.013975407137106616
```

In [80]:

```
analyzer.plot_move_inc_corr('Heilongjiang', use_best_fit=True)
```

```
offset = 4, window = 10, corr = 0.874190948725279
```

In [81]:

```
analyzer.plot_move_inc_corr('Xinjiang', use_best_fit=False)
```

```
offset = 0, window = 1, corr = 0.24886375322144283
```

In [82]:

```
analyzer.plot_move_inc_corr('Xinjiang', use_best_fit=True)
```

```
offset = 4, window = 10, corr = 0.7371702807900217
```

In [83]:

```
analyzer.plot_move_inc_corr('Guizhou', use_best_fit=False)
```

```
offset = 0, window = 1, corr = -0.10250522909665366
```

In [84]:

```
analyzer.plot_move_inc_corr('Guizhou', use_best_fit=True)
```

```
offset = 5, window = 10, corr = 0.8357913006722355
```

In [ ]:

```

```
